# Seryl-tRNA Synthetase Shows a Noncanonical Activity of Upregulating Laccase Transcription in *Trametes hirsuta* AH28-2 Exposed to Copper Ion

**DOI:** 10.1128/spectrum.00768-23

**Published:** 2023-07-03

**Authors:** Zhiwei Gan, Xueping Zhang, Mengke Li, Xing Li, Xinlei Zhang, Chenkai Wang, Yazhong Xiao, Juanjuan Liu, Zemin Fang

**Affiliations:** a School of Life Sciences, Anhui University, Hefei, Anhui, China; b Anhui Key Laboratory of Modern Biomanufacturing, Hefei, Anhui, China; c Anhui Provincial Engineering Technology Research Center of Microorganisms and Biocatalysis, Hefei, Anhui, China; Universita degli Studi del Molise

**Keywords:** seryl-tRNA synthetase, laccase, copper ion, yeast one-hybrid screen, transcription

## Abstract

The function of Seryl-tRNA synthetase in fungi during gene transcription regulation beyond translation has not been reported. Here, we report a seryl-tRNA synthetase, ThserRS, which can negatively regulate laccase *lacA* transcription in Trametes hirsuta AH28-2 under exposure to copper ion. ThserRS was obtained through yeast one-hybrid screening using a bait sequence of *lacA* promoter (−502 to −372 bp). *ThserRS* decreased while *lacA* increased at the transcription level in *T*. *hirsuta* AH28-2 in the first 36 h upon CuSO_4_ induction. Then, *ThserRS* was upregulated, and *lacA* was downregulated. *ThserRS* overexpression in *T*. *hirsuta* AH28-2 resulted in a decrement in *lacA* transcription and LacA activity. By comparison, *ThserRS* silencing led to increased LacA transcripts and activity. A minimum of a 32-bp DNA fragment containing two putative xenobiotic response elements could interact with ThserRS, with a dissociation constant of 919.9 nM. ThserRS localized in the cell cytoplasm and nucleus in *T. hirsuta* AH28-2 and was heterologously expressed in yeast. *ThserRS* overexpression also enhanced mycelial growth and oxidative stress resistance. The transcriptional level of several intracellular antioxidative enzymes in *T. hirsuta* AH28-2 was upregulated. Our results demonstrate a noncanonical activity of SerRS that acts as a transcriptional regulation factor to upregulate laccase expression at an early stage after exposure to copper ions.

**IMPORTANCE** Seryl-tRNA synthetase is well known for the attachment of serine to the corresponding cognate tRNA during protein translation. In contrast, its functions beyond translation in microorganisms are underexplored. We performed *in vitro* and cell experiments to show that the seryl-tRNA synthetase in fungi with no UNE-S domain at the carboxyl terminus can enter the nucleus, directly interact with the promoter of the laccase gene, and negatively regulate the fungal laccase transcription early upon copper ion induction. Our study deepens our understanding of the Seryl-tRNA synthetase noncanonical activities in microorganisms. It also demonstrates a new transcription factor for fungal laccase transcription.

## INTRODUCTION

Aminoacyl-tRNA synthetases (aaRSs) are a family of enzymes catalyzing the charging of amino acids onto their cognate tRNAs in the first step of protein synthesis throughout life kingdoms (1). In most species, the aaRS family contains 20 members, which can be classified into two classes based on the crystal structure and the site of the aminoacylated -OH group (1, 2). The class I enzyme family shares the conserved HIGH and KMSKS motifs in the active site, approaches the tRNA acceptor end from a minor groove, and aminoacylates the 2′-OH (or both the 2′-OH and 3′-OH) of an adenosine nucleotide. In contrast, the class II enzyme family shares three conserved sequence motifs comprising antiparallel β-sheets in their active site, approaches the tRNA acceptor end from the major groove, and aminoacylates the 3′-OH of an adenosine nucleotide (1).

Seryl-tRNA synthetase (SerRS) is a member of class II aaRSs (3, 4). It consists of at least two domains in all species, including the N-terminal tRNA-binding domain (TBD) and the globular catalytic domain (CD). In the aminoacylation reaction, CD mediates the dimerization interface and ensures that SerRS functions as a dimer. In particular, a seven-stranded antiparallel β-sheet in CD is critical in ensuring the activity. It provides a conserved motif comprising an α-helix followed by a distorted β-strand. It also contains a conserved Pro residue involved in homodimerization. Moreover, it contains another two motifs harboring conserved Arg residues that play a role in ATP binding (1, 5, 6). On the contrary, TBD is slightly conserved among SerRSs. Unlike other aaRSs identifying the anticodon, the TBD in SerRSs recognizes the long variable arm of tRNA^Ser^ (7–9). Moreover, a variable C-terminal extension presents in SerRSs from eukaryotes but not in prokaryotes, such as Escherichia coli and Bacillus subtilis. In yeast and maize, the C terminus of SerRS affects the stability and substrate affinity of itself (10, 11). Its truncation is toxic for Saccharomyces cerevisiae (10).

Independent of catalyzing the aminoacylation reaction, SerRSs from vertebrates play transcriptional roles in angiogenesis, cellular senescence, and lipid synthesis. In zebrafish, impairing SerRS or inhibiting vascular endothelial growth factor A (VEGFA) signaling leads to the aberrant branching of vessels (12). This phenomenon indicates a possible relationship between SerRS and VEGFA. Another two studies show that human SerRS can enter the nucleus, directly bind to the *vegfa* promoter, and interact with other transcription factors (13–15). In detail, it binds to the −62- to −38-bp region of the *vegfa* promoter and blocks c-Myc binding through direct head-to-head competition. Then, the DNA-bound SerRS recruits the SIRT2 histone deacetylase to erase prior c-Myc-promoted histone acetylation and regulates the proper development of functional vasculature (14). Furthermore, its CD can interact with the transcription factor yin-yang 1 (YY1) to form the SerRS/YY1 complex and negatively regulates *vegfa* by binding distal cis-regulatory elements at −4,654 to −4,623 bp to compete with the subunit of NF-κB (NFKB1) (15). For cellular senescence, SerRS directly binds to telomeric DNA repeats, interacts with protection of telomeres 1 (POT1), and tethers other POT1 proteins to prevent the recruitment of telomerase to telomeres, thus participating in telomere length control (16). Human SerRS can also suppress the key genes involved in the *de novo* lipid biosynthesis in the nucleus (17). A unique domain named UNE-S substituting the carboxyl terminus in SerRS is proposed to be responsible for these transcriptional functions in vertebrates. A robust nuclear localization signal (NLS) in UNE-S directs SerRS into the nucleus (6, 13–16, 18). However, the transcriptional regulatory role of SerRS in other kingdoms is not reported.

Laccase (benzenediol: oxygen oxidoreductases, EC1.10.3.2) is a type of multicopper oxidase that catalyzes the oxidation of hydroxyl functional groups in various substrates and the reduction of molecular oxygen into water (19, 20). It is present in all domains of life, including higher plants, insects, bacteria, and especially basidiomycete fungi (21, 22). As an essential ion for laccase activity, copper ion (Cu^2+^) is also a potent inducer that upregulates laccase expression at the transcriptional level (20, 23). Several transcriptional factors, including a copper-responsive transcription factor angiotensin-converting enzyme 1 (ACE1) and its homolog copper-dependent transcription factor 1 (CUF1) from Ceriporiopsis subvermispora (24), Polyporus brumalis (25), and Cryptococcus neoformans (26), an helix-turn-helix (HTH)-type protein leukotriene F4 (Ltf4) from Pleurotus ostreatus (27), a heat shock protein 70 (Hsp70) homolog Ssa1 in C. neoformans (28), and a heat shock transcription factor (HSF) 2 from Trametes trogii (29), are related to laccase transcription in response to Cu^2+^. Other transcription factors involved in laccase expression regulation upon Cu^2+^ induction remain unknown in all organisms.

Trametes hirsuta AH28-2 is a basidiomycete fungus isolated from rotting wood in China (30). Cu^2+^ at 1 to 2 mM can induce laccase LacA expression at the transcriptional level in a glucose medium (30, 31). In the present study, the yeast one-hybrid approach was used to mine the potential regulators participating in *lacA* transcription using a bait sequence amplified from the *lacA* promoter. A SerRS homolog, namely, ThserRS, was identified. ThserRS acted as a specific repressor upregulating *lacA* transcription early in response to Cu^2+^ by binding directly to a minimum of a 32-bp DNA fragment in the *lacA* promoter. We further demonstrated that *ThserRS* overexpression accelerated *T. hirsuta* AH28-2 colony growth upon stress because of its effect on the other antioxidative enzyme transcription.

## RESULTS

### Screening and verification of a candidate transcriptional factor interacting with *lacA* promoter.

**FIG 1:**
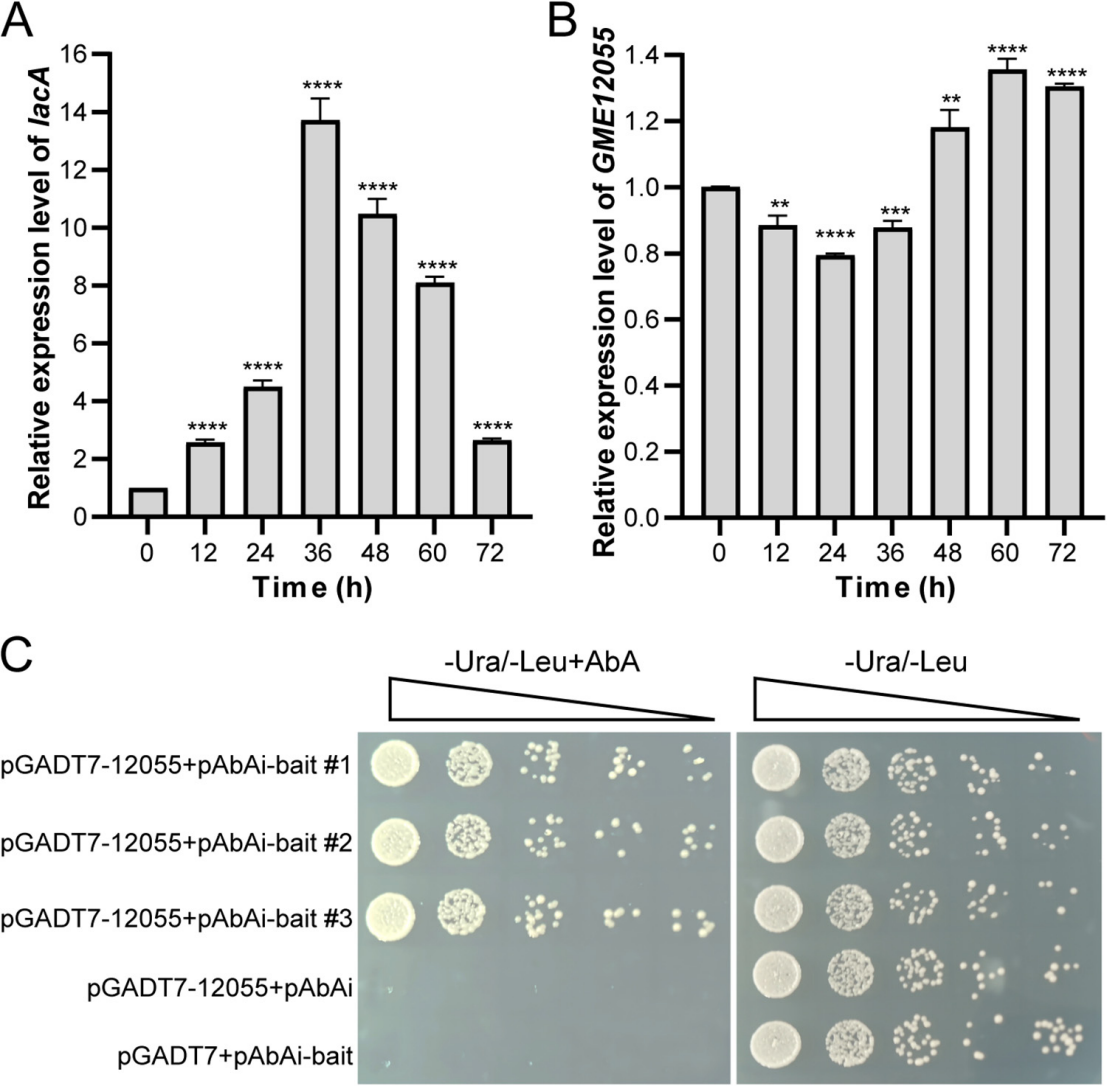
Verification of a candidate *lacA* promoter binding protein GME12055. (A and B) *lacA* is upregulated (A) but GME12055 is downregulated (B) in *T. hirsuta* AH28-2 upon Cu^2+^. *T. hirsuta* AH28-2 mycelia exposed to 100 μM CuSO_4_ were collected every 12 h and extracted for RNA, and the transcripts of *ThserRS* and *lacA* were analyzed by qRT-PCR. The transcriptional levels of *ThserRS* and *lacA* at 0 h were set as baselines. (C) GME12055 specifically interacts with *lacA* promoter in yeast. The pGADT7-12055 plasmid containing GME12055 cDNA was transformed into the Y1H-Bait reporter strain that inserted a bait DNA fragment (−502 to −372 bp) from the *lacA* promoter. The interaction of the bait DNA and GME12055 allowed yeast transformants to grow on SD/-Ura/-Leu/AbA^900^ plates. Controls either without the bait DNA (pGADT7-cDNA plus Y1H-pAbAi) or without GME12055 cDNA (pGADT7 plus Y1H-Bait) were used, respectively. The data were analyzed using Student’s *t* test (**, *P ≤ *0.01, ***, *P ≤ *0.001, or ****, *P ≤ *0.0001). Data show mean ± standard deviation; *n* = 3.

According to our previous study and the results presented in the present work, *lacA* was strongly induced to upregulation at the transcriptional level in the first 36 h after Cu^2+^ addition. This phenomenon resulted in a continuous increase in enzymatic activity at 24 h in *T. hirsuta* AH28-2 (Fig. 1A and see Fig. S1 in the supplemental material). To obtain transcription factors that participate in *lacA* transcription regulation in response to Cu^2+^, a 131-bp *lacA* promoter fragment containing two putative xenobiotic-responsive elements (XREs; TCACGC) and a metal-responsive element (MRE; TGCGCCC) was fused to an Aureobasidin A (AbA) resistance gene *AUR1-C* and used as the bait DNA based on the yeast one-hybrid approach. *T. hirsuta* AH28-2 mycelia exposure to 100 μM CuSO_4_ for 24 h was chosen for the cDNA library construction. This library was further transformed into the reporter strain, and transformants whose *AUR1-C* was expressed after the bait DNA activation were selected on plates containing 900 ng/mL AbA. After 2 rounds of screening, 158 potent clones out of 3 million transformants were picked, and the inserted cDNAs were amplified and sequenced (Fig. S2). Seventeen open reading frames were identified, of which three sequences were annotated to code for a putative SerRS (GME12055) and two putative glutathione *S*-transferases (GME2703 and GME4816).Reverse transcription-quantitative PCR (qRT-PCR) analysis showed that the transcriptional levels of GME2703 and GME4816 were upregulated significantly at 12 h in *T. hirsuta* AH28-2 upon Cu^2+^ induction (*P < *0.0001) and declined rapidly afterward (Fig. S3). In contrast, the GME12055 transcription level decreased in the first 24 h and increased afterward (Fig. 1B). The comparison of the transcriptional levels of GME12055 and *lacA* showed opposite variation trends, with a lag phase in between (Fig. 1A and B). Thus, GME12055 was chosen for further verification in S. cerevisiae. Furthermore, the positive transformants harboring the full-length cDNA of GME12055 grew on the SD/-Ura/-Leu/AbA^900^ plate. This finding suggested the specific recognition between GME12055 and the *lacA* promoter (Fig. 1C).

### Phylogeny and structure modeling.

**FIG 2:**
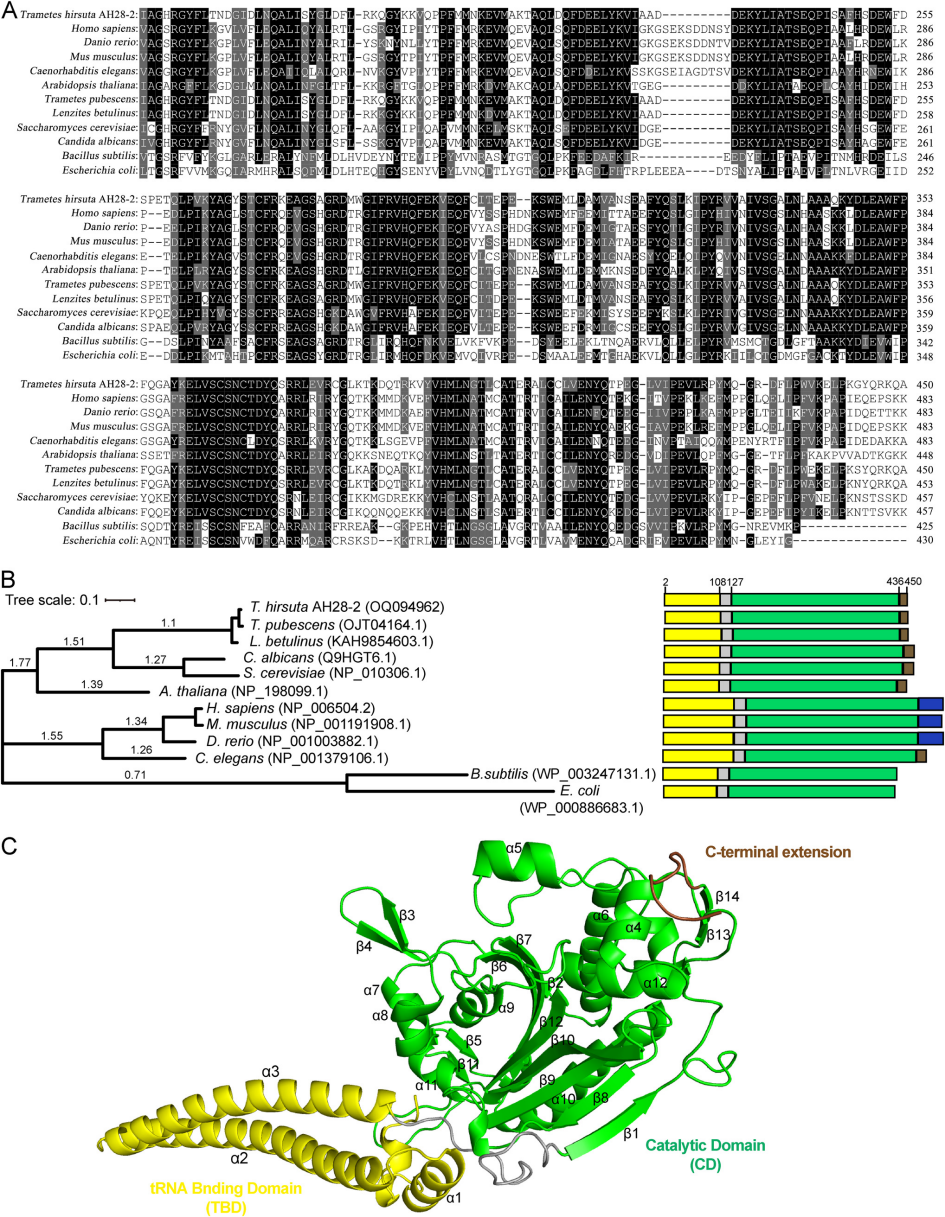
Phylogeny and structure modeling. (A and B) Amino acid alignment (A) and phylogenetic analysis (B) of ThserRS with its homologs from 11 other species. The phylogenetic tree in panel B was constructed using RaxML software based on the maximum likelihood method. (C) Structure modeling of ThserRS using human SerRS as the template. Yellow, gray, green, and brown boxes or residues in panels B and C indicate the tRNA-binding domain (TBD), the linker sequence (L), the globular catalytic domain (CD), and the C terminal, respectively. Blue boxes in panel B indicate the unique UNE-S domain in vertebrates.

GME12055 is 1,353 bp in length. It encoded a 450-amino-acid protein. The protein was annotated as a SerRS homolog and thus named ThserRS. Sequence alignment suggested that ThserRS shared sequence identity higher than 94% with SerRSs from Basidiomycetes, including Trametes versicolor, Trametes pubescens, and Lenzites betulinus. In addition, ThserRS had 58.93% and 61.21% sequence identities with the SerRS homologs in S. cerevisiae and Homo sapiens, respectively (Fig. 2A and B). Further analysis showed that SerRSs shared high sequence similarity among eukaryotes. However, they are distanced from the SerRSs in prokaryotes. For example, ThserRS shared approximately 35% of identities with those from E. coli and B. subtilis.

Structure modeling using human SerRS as the template revealed that ThserRS comprised two feature domains of the family, including TBD (2 to 108 AA), CD (127 to 436 AA), and a C-terminal extension (437 to 450 AA). ThserRS shared the comparable length of TBD and CD in amino acid sequence with SerRSs from fungi, but it was shorter than vertebrate homologs (Fig. 2B). As shown in Fig. 2C and Fig. S4, ThserRS contained two long α-helices (α2 and α3) with a short loop in between in the TBD and a seven-stranded antiparallel β-sheet (β1, β6, β7, β8, β9, β10, and β12) in the CD. A search of the *T. hirsuta* AH28-2 genome with the conserved domains of SerRS suggested only one SerRS homolog in this fungus. Furthermore, the C-terminal extension was relatively conserved among Basidiomycetes but variable from other eukaryotes. In *T. hirsuta* AH28-2, the C-terminal extension was relatively shorter than that formed by a unique domain, named UNE-S, in vertebrates (14 AA in length versus 45 AA from H. sapiens; Fig. 2B and C).

### ThserRS negatively regulates *lacA* transcription in *T. hirsuta* AH28-2 exposed to Cu^2+^.

**FIG 3:**
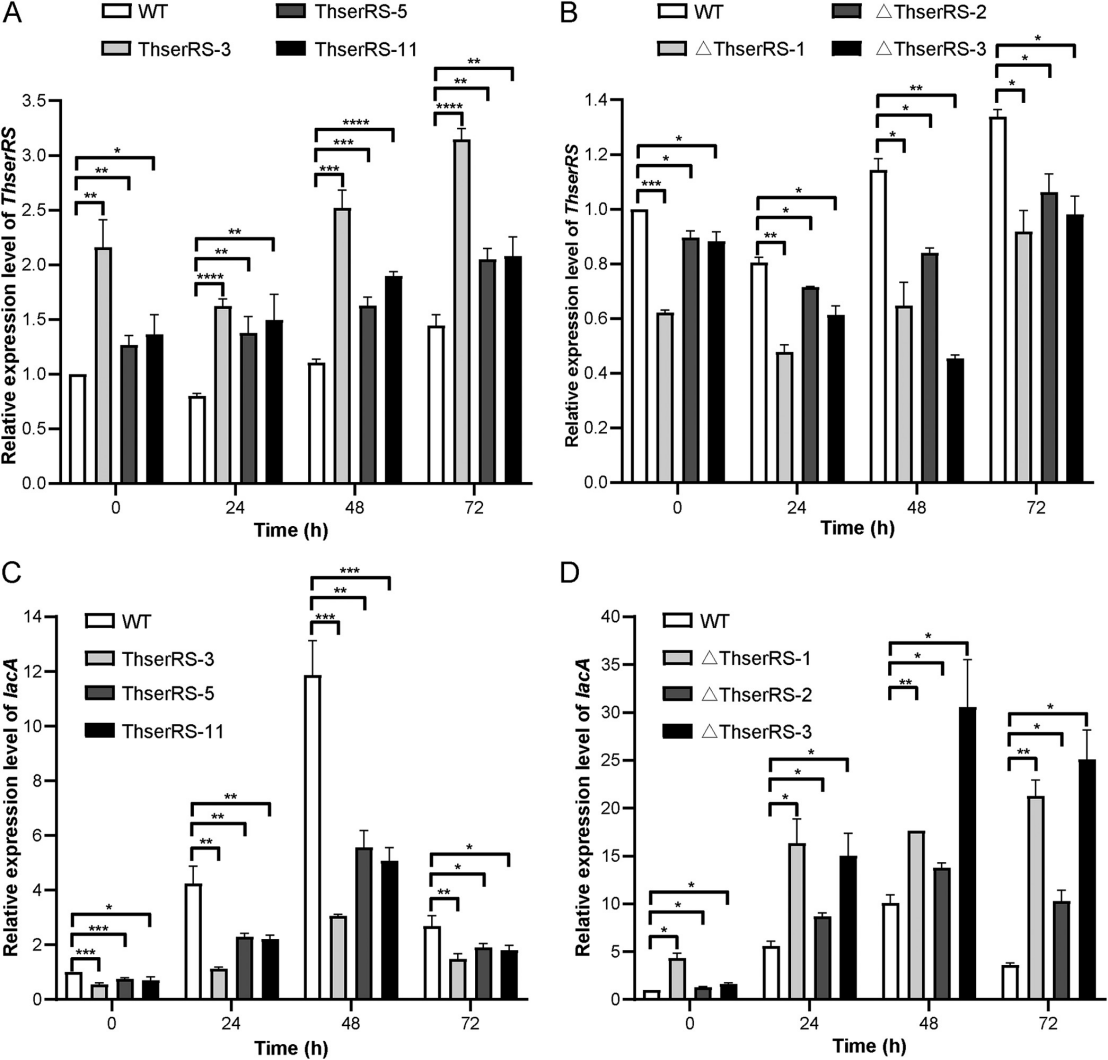
ThserRS negatively regulates *lacA* transcription upon Cu^2+^ induction. (A and B) *ThserRS* is upregulated in the overexpression strains (A) and downregulated in the silencing strains (B). *T. hirsuta* AH28-2 mycelia cultured in liquid XH medium with 100 μM CuSO_4_ added were collected every 24 h and extracted for RNA, and the transcriptional levels of *ThserRS* were analyzed by qRT-PCR analysis. The *ThserRS* transcriptional level of the wild-type strain (WT) at 0 h was set as the baseline. (C and D) *lacA* transcripts decreased in three *ThserRS* overexpression strains (C) and increased in three *ThserRS* silencing strains (D) shown by qRT-PCR analysis. The *lacA* transcriptional level of the WT at 0 h of 100 μM CuSO_4_ addition was set as the baseline. The data were analyzed using a Student’s *t* test (*, *P < *0.05, **, *P < *0.01, ***, *P < *0.001, ****, *P < *0.0001). Data show mean ± standard deviation; *n* = 3.

*ThserRS* overexpression and silencing strains were successfully constructed to verify whether ThserRS affected LacA expression in *T. hirsuta* AH28-2 upon Cu^2+^ induction. Eleven *ThserRS* overexpression transformants and six *ThserRS* silencing transformants were obtained based on hygromycin B selection and rescreening using PCR amplification of the *ThserRS* full-length or antisense fragment. Three overexpression strains, named ThserRS-3, ThserRS-5, and ThserRS-11, and three silencing strains, named ΔThserRS-1, ΔThserRS-2, and ΔThserRS-3, were randomly chosen for the qRT-PCR analysis. *ThserRS* transcripts increased 1.3 to 2.2-fold compared to the wild-type strain at 0 h and significantly upregulated during 0 to 72 h of Cu^2+^ addition in three overexpression strains (*P < *0.05, *P < *0.01, and *P < *0.001; Fig. 3A). On the contrary, the transcriptional levels of *ThserRS* in three silencing strains were downregulated during the cultivation, with 27% to 61% decrease at 48 h of Cu^2+^ addition (*P < *0.05 and *P < *0.01; Fig. 3B).

**FIG 4:**
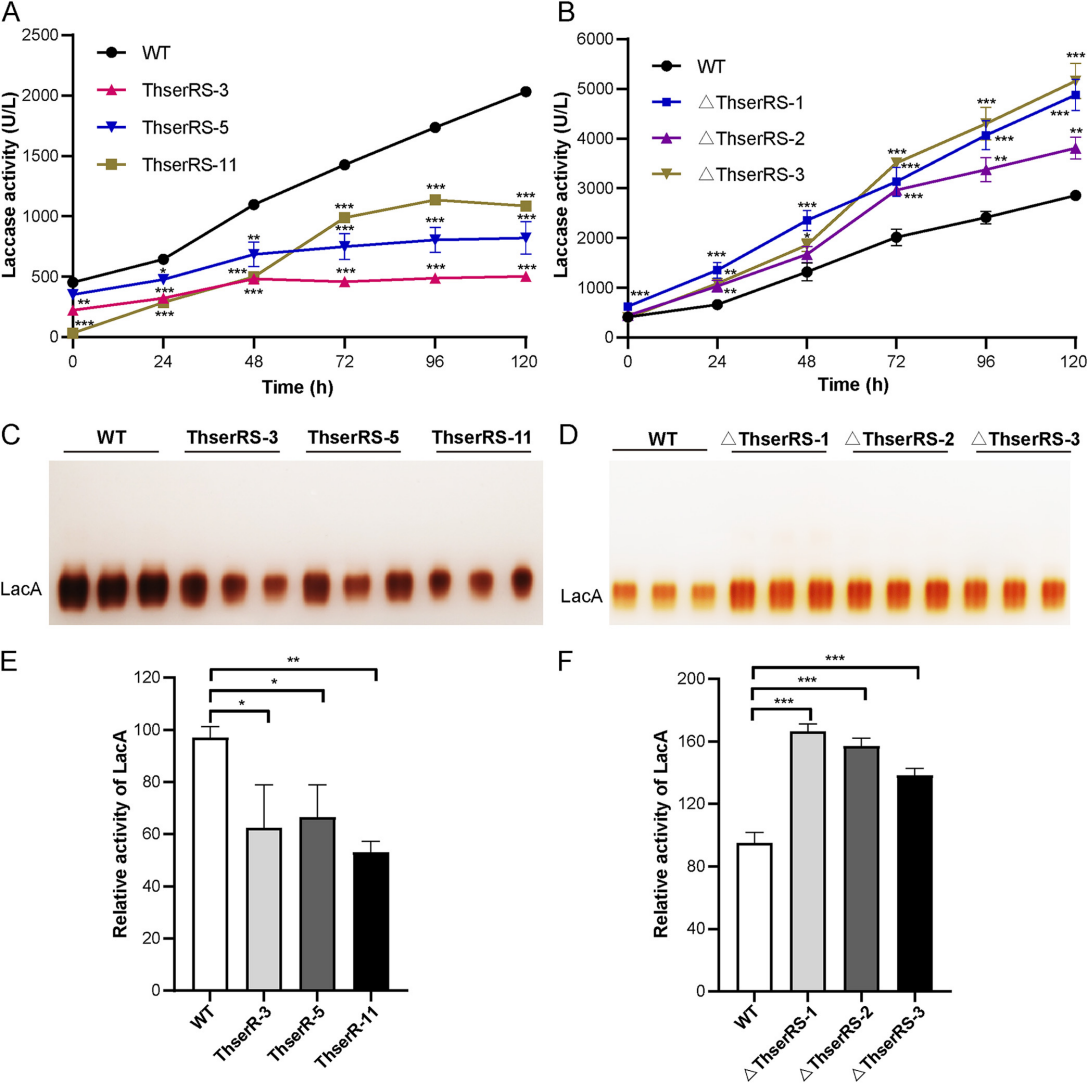
ThserRS negatively regulates LacA activity in *T. hirsuta* AH28-2 upon Cu^2+^ induction. (A and B) The volume laccase activity is downregulated by *ThserRS* overexpression (A) and upregulated by *ThserRS* silencing (B). (C and D) Native-PAGE of LacA activities at 48 h of 100 μM CuSO_4_ treatment of *T. hirsuta* AH28-2 indicate downregulation in *ThserRS* overexpression strains (C) and upregulation in *ThserRS* silencing strains (D). Equal concentrations of protein were loaded onto the gels for each sample and activity stained after gel electrophoresis. (E and F) Quantification of LacA activities deduced from the gels in panels C and D. The data were analyzed using a Student’s *t* test (*, *P < *0.05, **, *P < *0.01, or ***, *P < *0.001). Data show mean ± standard deviation; *n* = 3.

Compared with the wild-type strain, *ThserRS* overexpressing in *T. hirsuta* AH28-2 led to a 45% to 75% decrease in *lacA* transcription level throughout the cultivation (Fig. 3C). However, compared with the wild-type strain, *ThserRS* silencing strains resulted in increased *lacA* transcripts by 1.4- to 3.0-fold (Fig. 3D). Accordingly, the laccase activity of *ThserRS* overexpression strains reduced by more than 50% compared with that observed in the wild-type strain after 48 h of induction by Cu^2+^ (Fig. 4A). However, the laccase activity in *ThserRS* silencing strains was 1.3- to 1.8-fold higher than that in the wild-type strain (Fig. 4B). After the protein concentrations adjusted to consistency, native PAGE demonstrated that LacA activity was 35% to 46% decreased in *ThserRS* overexpression strains and 140% to 170% overexpressed in *ThserRS* silencing strains (Fig. 4C to F). These results demonstrated that ThserRS was a repressor for LacA expression in liquid fermentation when exposed to Cu^2+^ in *T. hirsuta* AH28-2.

### ThserRS binds directly to the *lacA* promoter *in vitro* and localizes in the nucleus.

**FIG 5:**
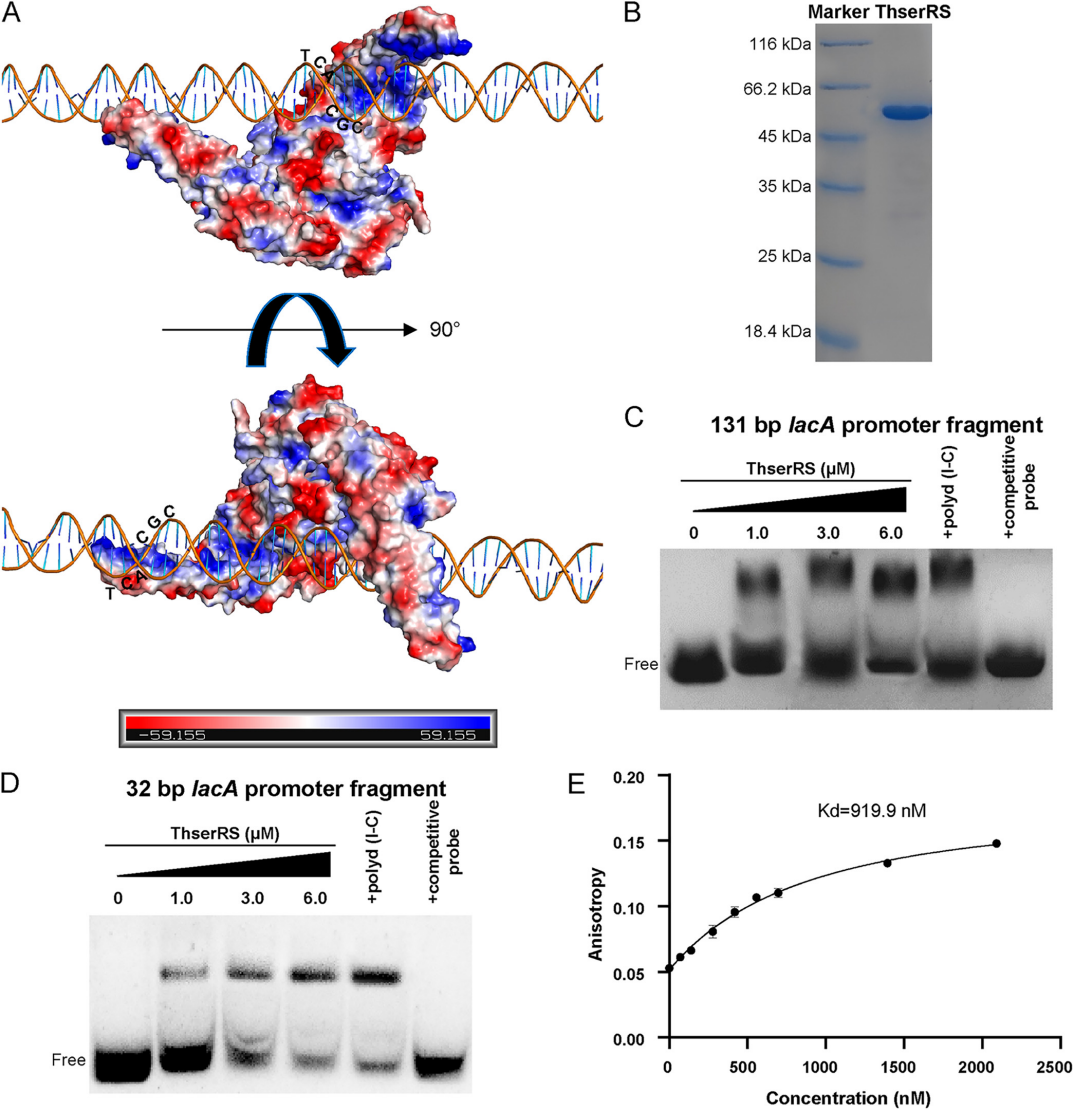
ThserRS directly binds with *lacA* promoter *in vitro*. (A) Structure modeling of ThserRS interacted with *lacA* promoter. A minimum of a 32-bp DNA fragment was bound with ThserRS. The residues in the two long α-helices (α2 and α3) in the TBD of one ThserRS monomer bound to one end of the DNA, while the residues in the short loop between α2 and α3 in the TBD of the other ThserRS monomer bound to the other end of the DNA. The residues in β6 of both ThserRS monomers interacted with the middle region of the DNA. (B) ThserRS is heterologously expressed in E. coli BL21(DE3) and purified. (C and D) The EMSAs show that ThserRS directly binds to the *lacA* promoter (−502 to −372 bp) (C) or the 32-bp minimum DNA fragment in *lacA* promoter (−462 to −431 bp) (D). One hundred nanograms of poly d(I-C) was added as the nonspecific competitor, and 10-fold identical unlabeled DNA probe was added as the specific competitor, respectively. (E), The binding of ThserRS and the 32-bp DNA probe shares a dissociation constant (*K_d_*) of 919.9 nM. Experiments were repeated at least three times, and representative results are shown.

The three-dimensional (3D) models of dimeric ThserRS with the 131-bp DNA probe from the *lacA* promoter were simulated using HDOCK to explore the specific binding sites of ThserRS in the *lacA* promoter sequence. As shown in Fig. 5A, the residues in two long α-helices (α2 and α3) in the TBD of one ThserRS monomer bound to one end of the DNA, whereas the residues in the short loop between α2 and α3 in the TBD of the other ThserRS monomer bound to the other end of the DNA. Furthermore, the residues in β6 of both ThserRS monomers interacted with the middle region of the DNA. The DNA fragment in the *lacA* promoter interacting specifically with ThserRS was from −462 bp to −431 bp, harboring two XREs. One XRE interacted with the short loop of one ThserRS TBD (65 to 84 AA) and the other XRE interacted with the second ThserRS α3-helix and β6-sheet. Other nucleotides in this DNA fragment were also involved in this binding.

ThserRS was successfully expressed in E. coli BL21(DE3) and purified for electrophoretic mobility shift assay (EMSA; Fig. 5B). The results showed that a band shifted after the ThserRS was incubated either with a *lacA* promoter fragment tagged with 6-carboxyfluorescein (6-FAM; comprised of −502 bp to −372 bp) (Fig. 5C) or with the 6-FAM-tagged truncated 32-bp DNA probe (Fig. 5D). ThserRS could specifically bind to these two DNA probes because poly d(I-C) addition did not affect the shifted band. However, adding a 10-fold nonlabeled DNA fragment prevented the chemiluminescent complex formation (Fig. 5C and D). Fluorescence polarization (FP) assays further calculated a dissociation constant (*K_d_*) of 919.9 nM for the binding of ThserRS to the 32-bp DNA probe (Fig. 5E). More nucleotide truncations in the DNA probe failed in forming a stable complex in EMSA and FP experiments. Thus, a minimum of a 32-bp DNA fragment in the *lacA* promoter was involved in interacting with ThserRS *in vivo*.

**FIG 6:**
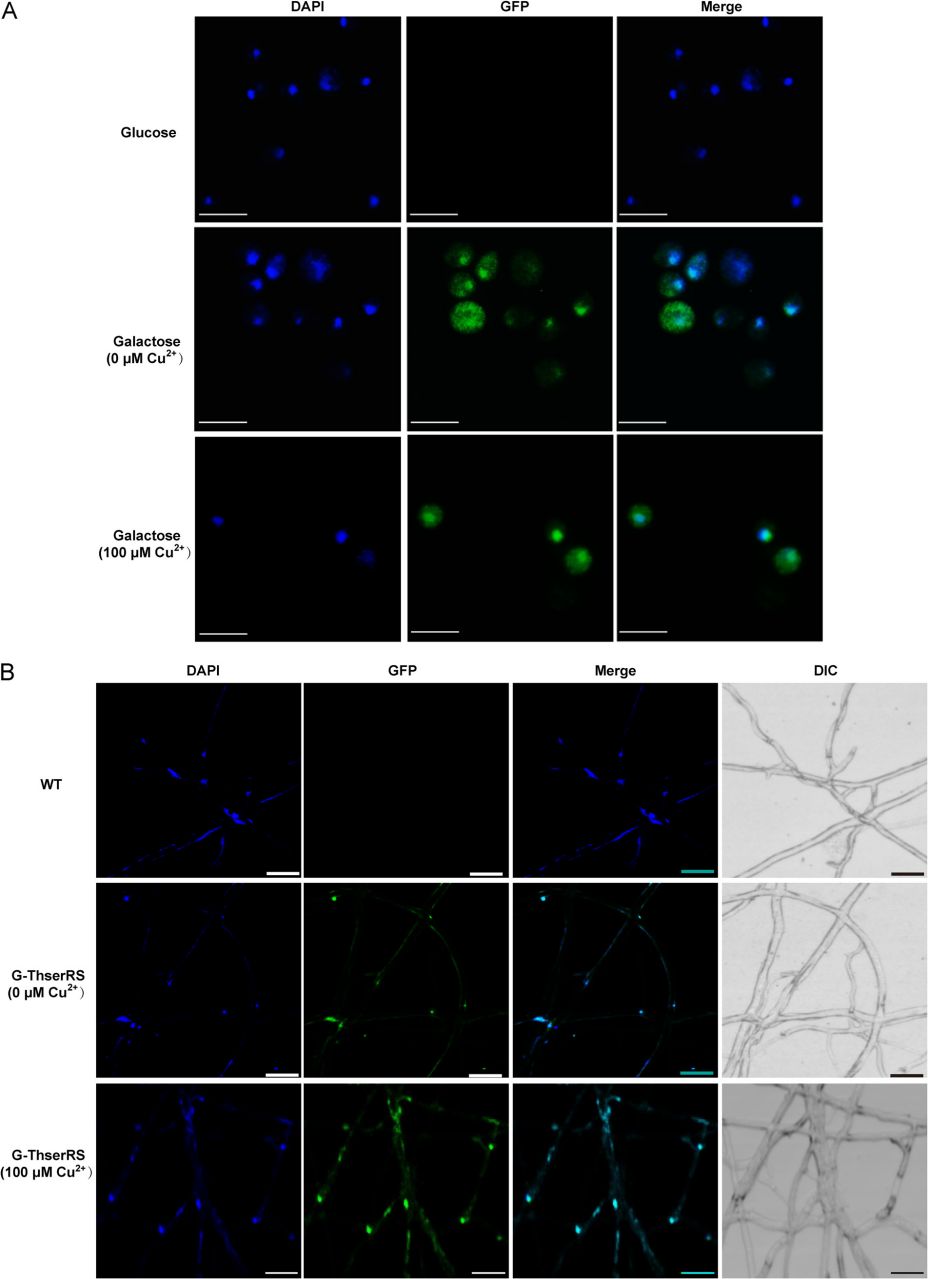
ThserRS is partially localized in nucleus in yeast and *T. hirsuta* AH28-2. (A) Y1H-GFP-ThserRS strain was spotted either on an SD-glucose plate or an SD-galactose plate with or without 100 μM CuSO_4_, incubated at 30°C for 3 days, and then stained with DAPI. (B) The *gfp*-*ThserRS* overexpression *T. hirsuta* AH28-2 mycelia were grown on microscope slides with XH agar medium, fixed with cold methanol, and then stained with DAPI. GFP-ThserRS is shown in green, and DAPI staining of the nucleus is shown in blue. Experiments were repeated at least three times, and representative results are shown. Scale bars = 10 μm.

Furthermore, a Y1H-GFP-ThserRS strain expressing GFP-ThserRS under the control of a galactose-inducible promoter was constructed to determine whether ThserRS could localize in the nucleus. GFP-ThserRS was not expressed in the control strain induced by glucose (Fig. 6A and Fig. S5). After GFP-ThserRS was triggered to express by galactose, fluorescence appeared in the whole cytoplasm, with higher fluorescence intensity in the nucleus either with or without Cu^2+^ induction. GFP-ThserRS was further overexpressed in *T. hirsuta* AH28-2 to determine its subcellular localization. The GFP fusion did not interfere with the function of ThserRS on *lacA* transcription in strain G-ThserRS (Fig. S6). Fluorescence results showed that GFP-ThserRS could partially accumulate in the nucleus without Cu^2+^ treatment. This phenotype hardly changed when exposed to Cu^2+^ (Fig. 6B). These results demonstrated that ThserRS could enter the nucleus and play roles as a nuclear protein under different culture conditions.

### ThserRS overexpression accelerates mycelia growth and increases oxidation tolerance.

**FIG 7:**
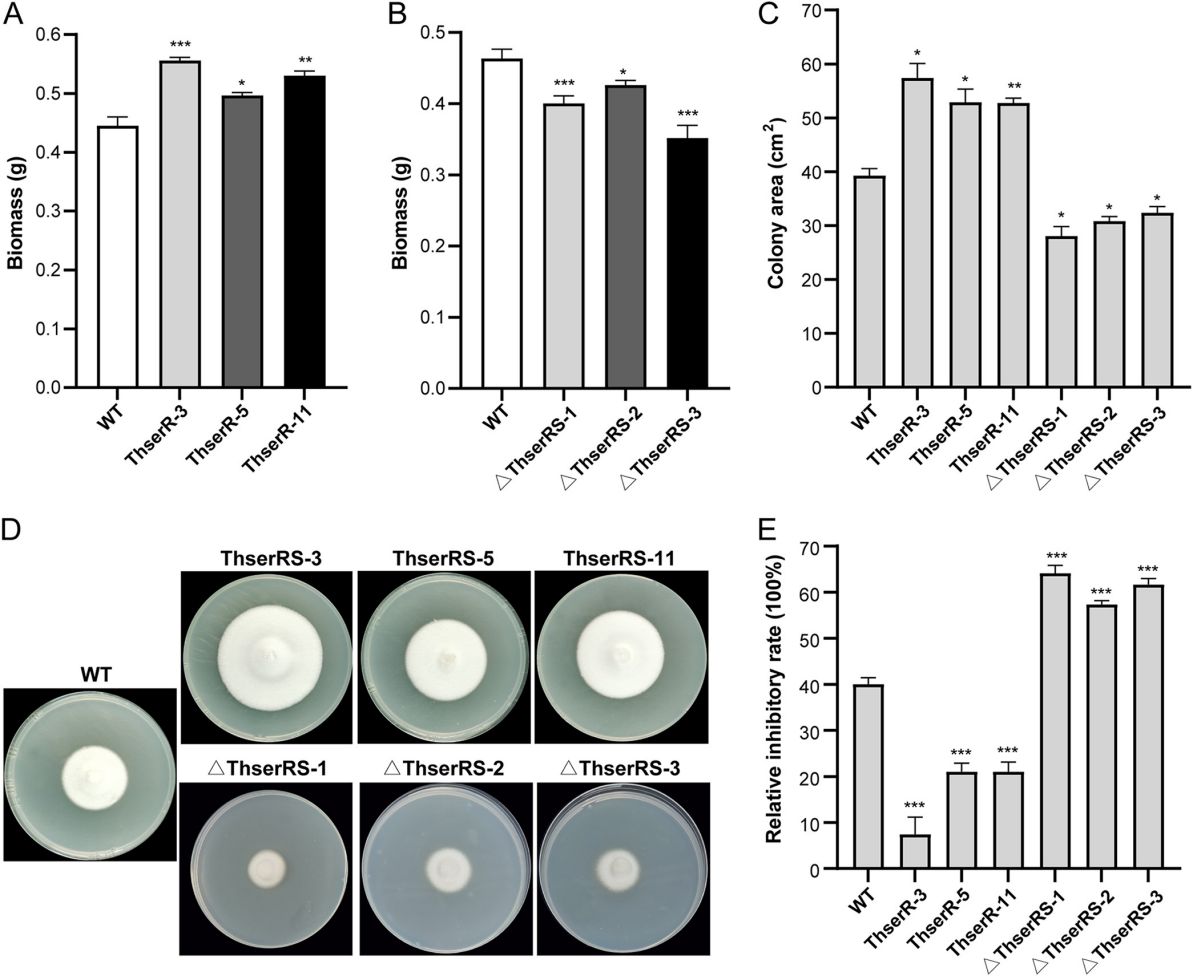
ThserRS is involved in mycelia growth and oxidation tolerance. (A and B) The biomass of the wild-type (WT), *ThserRS* overexpressing, and *ThserRS* silencing *T. hirsuta* AH28-2 strains. *T. hirsuta* AH28-2 mycelia cultured in liquid XH medium with 100 μM CuSO_4_ added were collected at 120 h. (C) Colony area was increased by *ThserRS* overexpression but decreased by *ThserRS* silencing when incubated on XH agar plates with 100 μM CuSO_4_ added for 5 days in a dark incubator at 28°C. (D) *ThserRS* overexpression strains show less sensitivity, but *ThserRS* silencing strains show more sensitivity to H_2_O_2_ than WT. Strains grown on XH agar plates with 30 mM H_2_O_2_ plates added are shown. (E) The growth inhibition rate was calculated from panel D (inhibition rate = [the diameter of untreated strain – the diameter of 30 mM H_2_O_2_-treated strain]/[the diameter of untreated strain] ×100%) (29). The data were analyzed using a Student’s *t* test compared with the WT strain (*, *P < *0.05, **, *P < *0.01, or ***, *P < *0.001). Data show mean ± standard deviation; *n* = 3.

Given that ThserRS is indispensable for protein translation, the mycelia of the wild-type, *ThserRS* overexpression, and silencing *T. hirsuta* AH28-2 strains were withdrawn at 120 h to evaluate the effect of ThserRS on mycelial growth under Cu^2+^ induction. The biomass increased in all ThserRS overexpression strains (*P < *0.05, *P* < 0.01, and *P < *0.001), whereas it decreased in silencing strains significantly (*P < *0.05 and *P < *0.001; Fig. 7A and B). The cultivation of strains on the XH agar plates containing Cu^2+^ also showed that *ThserRS* overexpression accelerated (*P < *0.05 and *P < *0.01); however, *ThserRS* silencing significantly reduced the mycelial expansion rate (*P < *0.01; Fig. 7C). In addition, the colony showed thin and wrinkled mycelial morphologies as the silencing strains grew (Fig. S7A). However, without Cu^2+^ induction, the biomasses in liquid media and the mycelial expansion rate on agar plates between the wild-type, *ThserRS* overexpression, and silencing *T. hirsuta* AH28-2 strains were almost similar to those exposed to Cu^2+^ (Fig. S7B and C). Thus, ThserRS was important for mycelial growth in *T. hirsuta* AH28-2 under different culture conditions, and the strains showed defective growth without sufficient ThserRS.

The overexpressed fungal laccase is suggested to facilitate mycelial growth and eliminate oxidative stress (32). The above seven strains were further exposed to oxidative stress raised by Cu^2+^ and H_2_O_2_. Surprisingly, ThserRS overexpression strains showed lower sensitivity than the wild-type strain toward extracellular oxidative stress on agar plates (Fig. 7D and E and Fig. S8). In contrast to a 40% reduction in the growth rate of the wild-type strain, 7%, 21%, and 21% reductions in three *ThserRS* overexpression strains were obtained by the treatment with 30 mM H_2_O_2_ (Fig. 7D and E). Under this treatment, the growth rate of the three *ThserRS* silencing strains decreased by 64%, 57%, and 62%, respectively (Fig. 7D and E). The mycelial density was considerably reduced and wrinkled.

**FIG 8:**
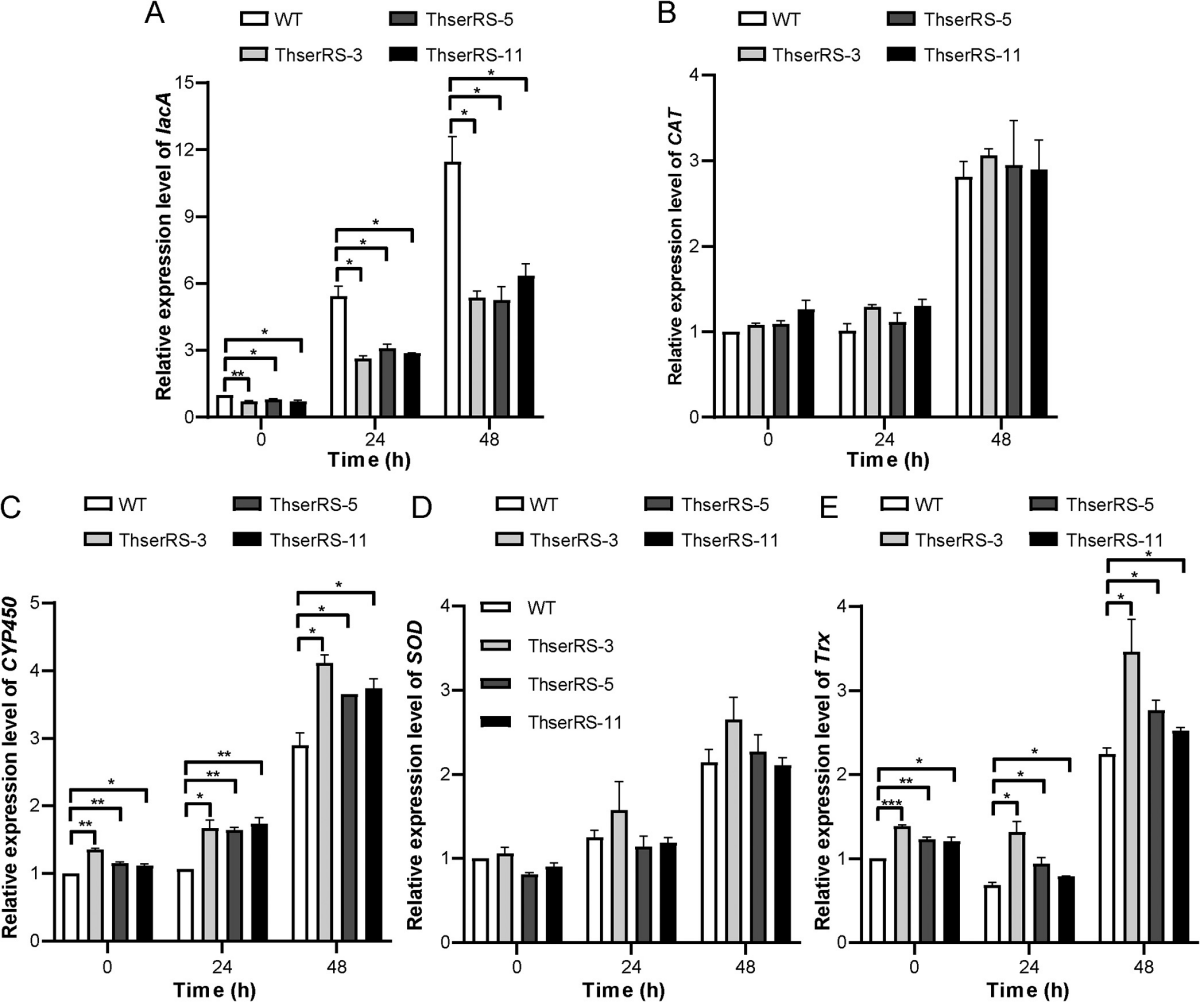
ThserRS differentially regulates antioxidant genes transcription upon oxidative stress. *T. hirsuta* AH28-2 mycelia cultured in liquid XH medium with 30 mM H_2_O_2_ added were collected at 0, 24, and 48 h and extracted for RNA, and the transcriptional levels of *lacA*, *Trx*, *SOD*, *P450*, and *CAT* were analyzed by qRT-PCR analysis. The transcriptional level of each gene in the wild-type strain (WT) at 0 h was set as the baseline. The data were analyzed using a Student’s *t* test (*, *P < *0.05, **, *P < *0.01, or ***, *P < *0.001). Data show mean ± standard deviation; *n* = 3.

Four representative antioxidant genes harboring XREs in promoters, including thioredoxin (*Trx*), superoxide dismutase (*SOD*), cytochrome P450 (*CYP450*), and catalase (*CAT*), as well as *lacA*, were further chosen for qRT-PCR analysis. Similar to *lacA*, the conserved sequence of XREs present in *CYP450* and *Trx* is TCACGC, whereas the conserved sequences of XREs present in *SOD* and *CAT* are TNGCGTG and TGCGTG, respectively (Table S2). In the wild-type strain, the *lacA* transcripts significantly increased within 24 h in the presence of Cu^2+^ and H_2_O_2_ (*P < *0.01), whereas the four intracellular enzymes were upregulated only after 24 h. Compared with the negative regulatory effect of ThserRS on *lacA* transcription, the effect of ThserRS overexpression on the *CYP450* and *Trx* transcripts was positive. However, the transcriptional levels of *CAT* and *SOD* were almost unchanged (Fig. 8).

## DISCUSSION

SerRS is one of the most ancient protein families. Independent of catalyzing the aminoacylation reaction, the functionalities of SerRSs expand gradually during evolution with the addition of new domains and motifs. In vertebrates, the genes involved in angiogenesis, cellular senescence, and lipid synthesis are regulated by SerRSs at the transcriptional level (6, 13–16, 18). However, the transcriptional regulatory role of SerRSs in other kingdoms of life has not been reported. In the present paper, we reported that a SerRS homolog, ThserRS, could localize in the nucleus and bind to a minimum of a 32-bp DNA fragment containing two putative XREs to regulate laccase LacA transcription in the basidiomycete fungus *T. hirsuta* AH28-2 in response to Cu^2+^.

According to the global transcriptional analysis, ThserRS transcripts changed throughout Cu^2+^ induction along with *lacA* transcripts with an opposite trend (Fig. 1 and Fig. S3). Afterward, ThserRS overexpression sharply downregulated the *lacA* transcription. At the same time, its silencing upregulated the LacA expression (Fig. 3 and 4). Furthermore, the results from structure simulation, EMSA, and FP assays suggested the direct interaction of ThserRS with the *lacA* promoter region (Fig. 5). These phenomena indicate that ThserRS could regulate *lacA* transcription by directly binding to its promoter (Fig. 5). Thus, for the first time, we showed that SerRS functions as a transcription factor to regulate laccase expression in fungi. The function beyond the translation of SerRS has not been developed except for vertebrates. However, increasing evidence suggests that in early evolution, selective pressures may endow aaRSs for other roles (6). For example, E. coli alanyl-tRNA synthetase represses its gene transcription by binding specifically to a palindromic sequence that flanks the gene’s transcription start site (33). The mitochondrial tyrosyl-tRNA synthetase in Neurospora crassa (34) and the mitochondrial leucyl-tRNA in S. cerevisiae (35) participate in the splicing of group intron-containing pre-mRNAs. Thus, the complexity and diversity of the nontranslation functions of SerRSs in different species need further investigation.

Proteins that need to be transported into the nucleus necessitate the presence of an NLS on them to be recognized by the corresponding nuclear transporters for reaching the nucleus through the nuclear pore complex (36). Beyond containing two domains of TBD and CD, vertebrate SerRS has a unique UNE-S domain harboring an NLS for entering the nucleus to participate in gene transcriptional regulation (13–15). However, no classical NLS was found in the three regions of ThserRS. Only the peptide AAKKKAKE_65–72_ in the short loop between α2 and α3 of TBD (Fig. S4) was proposed to be a probable NLS based on NLStradamus prediction but with a low score (37). Unfortunately, the deletion of this region resulted in a misfolded protein (data not shown). The mechanism that directed ThserRS into the nucleus was unknown. However, the results that the plasmid containing the *ThserRS* cDNA interacted with the bait DNA (Fig. 1) and the GFP-ThserRS partially colocalized with the nucleus marked with 4,6-diamidino-2-phenylindole (DAPI) (Fig. 6) suggested that ThserRS was indeed present in the nucleus. In fact, many nuclear proteins have nonclassical NLS or are not rich in arginine or lysine residues, such as the RG/RGG region (38–41). Furthermore, the revealed nuclear pore complex structure and the report that mechanical force affects the nuclear translocation of several transcriptional regulators also illustrate the complexity of the translocation process from the cytoplasm into the nucleus (42, 43).

Laccase is usually triggered or induced to overexpress at the transcriptional level in fungi during their confrontation with various environmental factors (23, 44–46). The cis-acting elements, such as carbon catabolite repressor, nitrogen repression response element, and MRE in the laccase promoter region, have been postulated to play roles in carbon, nitrogen, and metal ion response, respectively (20, 23, 47). However, the mechanisms controlling fungal laccase overexpression are poorly understood. Moreover, the regulation mechanism of Cu^2+^ on laccase transcription was partially explored in laboratory works and was mainly related to MRE/AceI (24–26). According to HDOCK simulation, EMSA, and FP experiments (Fig. 5), ThserRS could directly and specifically bind to a 32-bp DNA fragment containing two putative XREs (TCACGC) consisting of a conserved motif of T/GCG/AT/CGC/G (48). Both XREs were involved in this binding. This DNA binding sequence differs from the only reported one (GGGCGGAGCCATGCGCCCCCC) in the *VEGFA* promoter region interacting with human SerRS and containing a nonclassical enhancer box (CATGCG) (14).

XREs are responsible for recognizing exogenous environmental pollutants, which can cause damage to human cell growth and development, such as alcohols, acids/ketones, and drugs, controlling the inducible expression of many antioxidative enzymes (49, 50). They are present in fungal laccase gene promoters and are postulated to control laccase transcription upon exposure to xenobiotic-induced stress, such as aromatic compounds (30, 48, 51, 52). However, this observation has not been proven until now in laboratory work. As an essential ion for fungal growth and an inducer for laccase expression, a great deal of Cu^2+^ may also be xenobiotics for fungi, resulting in the response of XREs. In addition, Cu^2+^ can lead to oxidative stress (53). Thus, the cis-acting elements involved in stress responses may be triggered. Following this hypothesis, recent research has demonstrated that an HSF binds to a heat shock-responsive element of the laccase gene in *T. trogii* upon Cu^2+^ induction (29). Furthermore, Cu^2+^ is taken up by fungi through high-affinity transmembrane copper transporters (54). Different nitrogen conditions in the media or copper transporter gene deletion, which first affects these transporters’ expression and further regulates laccase gene transcription, also illustrate the complexity of laccase expression when treated with Cu^2+^ (20, 55). The copper-responsive signal pathway, the cis-acting elements, and their corresponding transcription factors remain underexplored and expanded.

Fungi have evolved promising strategies to come across environmental changes. In our study, *lacA* was promptly upregulated during the early stage after combining with CuSO_4_ and H_2_O_2_ in *T. hirsuta* AH28-2. Thus, LacA was proposed to secrete into the medium to relieve extracellular oxidative stress (32). In addition, several intracellular defense mechanisms have evolved to scavenge intracellular reactive oxygen species (ROS) (56). In the wild-type strain, SOD and CAT increased with the extension of exposure time to neutralize the ROS generated by Cu^+^ toxicity (20). The upregulated CYP450 participating in many metabolic reactions uses H_2_O_2_ as an oxygen-donating cosubstrate (27, 57). These downstream genes are regulated by many kinds of transcription factors (58, 59). The transcriptional levels of *CYP450* and *Trx* increased after overexpressing *ThserRS* suggesting positive regulation of ThserRS on *CYP450* and *Trx* but not *CAT* or *SOD*. Although the regulation mechanism remains under exploration, the fact that only the promoters of *CYP450* and *Trx* harbored the same XRE sequence (TCACGC) as *lacA* (Table S2) may provide clues on it. On the other hand, the XRE sequences found in *SOD* and *CAT* promoter sequences were TNGCGTG and TGCGTG, indicating that other transcription factors might regulate the two genes during mycelia confrontation. Thus, these four enzymes may act synergistically with LacA during stress defense.

The traditional physiological role of ThserRS is to participate in protein translation and provide protein synthesis flux. Following this fact, the present work shows that ThserRS is very important for *T. hirsuta* AH28-2 growth since *ThserRS* silencing led to growth defects whether or not the strain was confronted with Cu^2+^ (Fig. 7 and Fig. S7). Similar to our results, the disruption of *serRS* leads to no cellular viability in the Basidiomycetes smut fungus Ustilago maydis (60). Furthermore, the length of the hyphal-tip cells of *ThserRS* silencing strains was much shorter than the wild-type strain.

Simultaneously, our results suggest that ThserRS is a transcriptional factor in *T. hirsuta* AH28-2, leading us to rethink the physiological role of ThserRS. Because Ser is a substrate for Cys and Gly synthesis and laccase is a luxurious gene in basidiomycete, we proposed that the massive expression of laccase consumes an adequate amount of Ser in the cytoplasm (Ser comprises 6.93% of the total amino acids in LacA), restricting the synthesis of other proteins. Thus, ThserRS negatively controls LacA expression when unnecessary to maintain the dynamic balance of intracellular serine supply.

**FIG 9:**
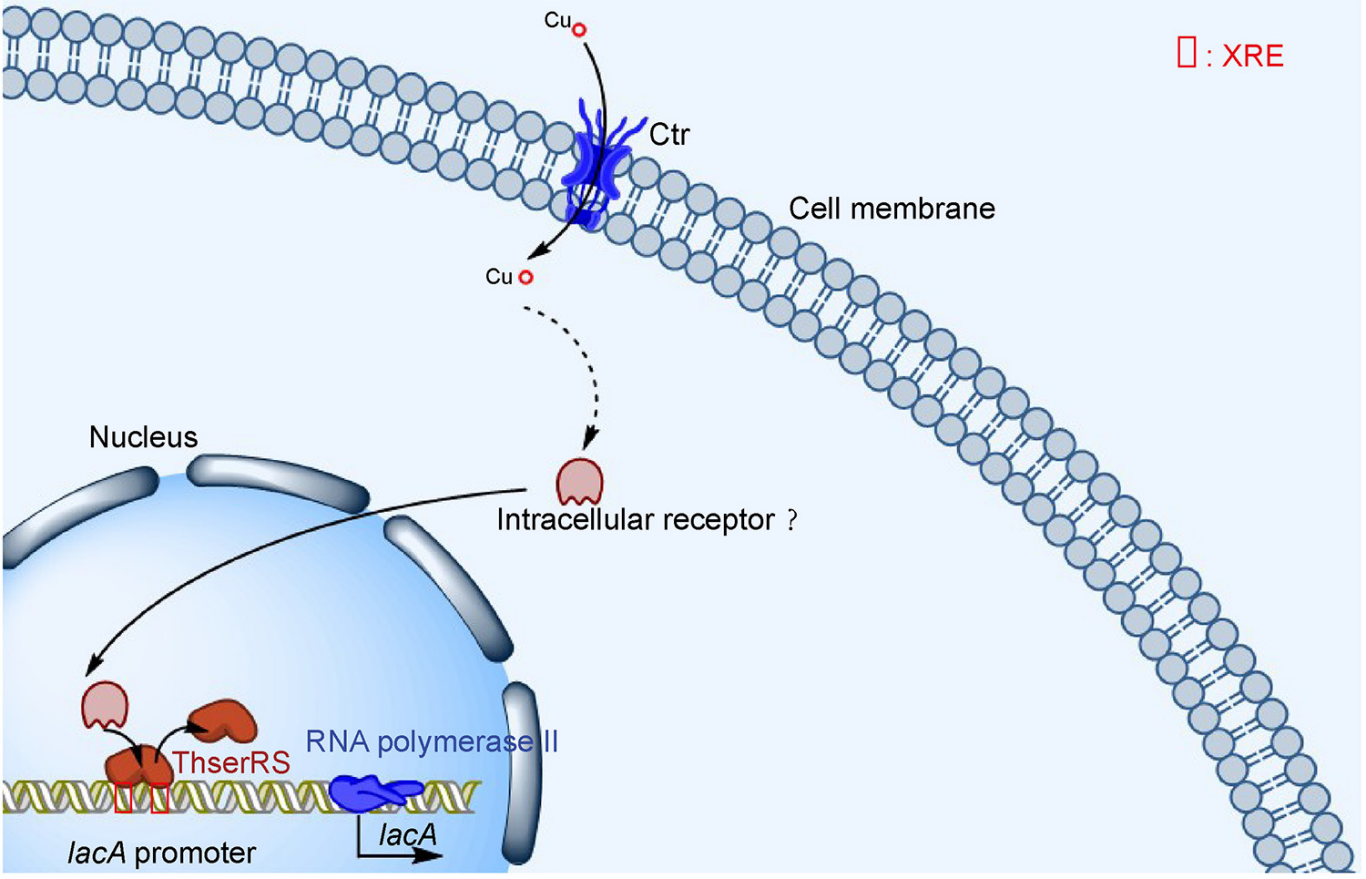
Model for the regulation mechanism of ThserRS on *lacA* transcription upon Cu^2+^ induction. The extracellular Cu^2^**^+^** is transported into the cytoplasm by high-affinity copper transporters (Ctr) and further mediates the activation signal into the nucleus by a putative intracellular receptor (54). The nuclear ThserRS can interact with *lacA* promoter and act as a negative regulator to increase *lacA* transcription rapidly.

In summary, for the first time, we demonstrated that ThserRS has a nonclassical role in *T. hirsuta* AH28-2 to respond to Cu^2+^ and negatively regulate laccase transcription (Fig. 9). Nuclear ThserRS interacts with the promoter of *lacA* and rapidly increases the transcription of *lacA* in response to Cu^2+^. A minimum of a 32-bp DNA fragment in the *lacA* promoter is involved in the interaction. *ThserRS* overexpression in the later stage upon Cu^2+^ induction and oxidative stress affects the transcription of other antioxidative enzymes. It also results in excellent resistance and a high growth rate in *T. hirsuta* AH28-2.

## MATERIALS AND METHODS

### Strains and culture media.

Strain *T. hirsuta* AH28-2 (China Center for Type Culture Collection No. AF 2015027) was maintained on the plates of compound potato dextrose agar (CPDA; per liter, filtrate of 200 g boiled potato, 20 g glucose, 3 g KH_2_PO_4_, 1.5 g MgSO_4_-7H_2_O, 0.05 g vitamin B_1_, and 15 g agar) at 4°C. The XH medium (per liter, 15 g cellobiose, 1 g peptone, 1.5 g dl-asparagine, 0.1 g Na_2_HPO_4_, 1 g KH_2_PO_4_, 0.5 g MgSO_4_-7H_2_O, 0.01 g CaCl_2_, 1 mg FeSO_4_-7H_2_O, 28 mg adenine, 0.05 mg vitamin B_1_, and 2 mg CuSO_4_-7H_2_O) was used for its liquid culture as previously described (46). CuSO_4_ was added at a final concentration of 100 μM to induce laccase expression.

E. coli DH5α, E. coli BL21(DE3), and E. coli HST08 were used for plasmid construction, heterologous ThserRS expression, and library cDNA transformation, respectively. All three strains were cultured in lysogeny broth (per liter, 20 g peptone, 10 g yeast extract, and 20 g glucose). In addition, the S. cerevisiae Y1H (Y1H; *MAT*α, *ura3-52*, *his3-200*, *ade2-101*, *trp1-901*, *leu2-3*, *112*, *gal4Δ*, gal80Δ, *met-*, and *MEL1*) maintained on the yeast peptone dextrose adenine medium (per liter, 10 g yeast extract, 20 g peptone, 20 g glucose, and 0.12 g adenine) was used as the host for the one-hybrid screening.

### Fungal submerged cultures.

Six actively growing *T. hirsuta* AH28-2 blocks (5 mm in diameter) on the plates were inoculated into liquid XH medium and continuously shaken at 120 rpm in the dark for 4 days. After homogenization, primary cultures (5%, vol/vol) were inoculated into 100 mL of fresh XH medium. They were precultured at 120 rpm with shaking for 72 h (30, 46) before 100 μM CuSO_4_ was added. This time point was defined as 0 h for induction. The cultures without added agents were used as controls. The experiments were performed three times, always with triplicate cultures per test case.

### *T. hirsuta* AH28-2 cDNA library construction.

The total RNA of *T. hirsuta* AH28-2 mycelia that were treated with 100 μM CuSO_4_ for 24 h was extracted using RNAiso Plus (TaKaRa, Japan). It was further digested with DNase I (TaKaRa, Japan) to remove the genomic DNA. Then, 1 μg of total RNA was used as the template for cDNA synthesis based on the Clontech Smart cDNA Library Construction kit (Clontech, USA) and Advantage 2 PCR kit (Clontech). cDNA was further homogenized using the Trimmer Direct cDNA Normalization kit (Evrogen, Russia) according to the manufacturer’s instructions. It was subsequently used for amplification to obtain homogenized cDNA. After purification, cDNA was dissolved in dH_2_O and digested with SfiI. The enzymatically cleaved cDNA was passed through a Chroma Spin-1000-TE column (Clontech) to remove the short fragments. Then, it was purified, refined in ethanol, and dissolved in dH_2_O. A primary cDNA library was constructed by ligating the cDNA fragments into the pGADT7-SfiI vector according to the Matchmaker Gold Yeast One-Hybrid Screening System user manual (Clontech). Then, it was transformed into competent E. coli HST08 cells to generate the library plasmid.

### Mining potential regulators from *T. hirsuta* AH28-2 cDNA library.

A bait DNA fragment from the *lacA* promoter (−502 bp to −372 bp) was amplified using the primer pair Bait-F and Bait-R (Table S1) and cloned into the pAbAi vector (Clontech) containing the AbA resistance gene *AUR1-C* at *Sac*I and *Sal*I sites to generate the reporter plasmid pAbAi-Bait. This plasmid was integrated into the Y1H genome. The generated Y1H-Bait reporter strain was screened on synthetic defined (SD) plates (per liter, 6.7 g YNB, 20 g glucose, 1% vol/vol dropped-out supplement) without uracil but with 900 ng/mL AbA (SD/-Ura/AbA^900^ plates). Then, PCR validation followed using primers pAbAi-F and pAbAi-R in Table S1. The SmaI linearized pGADT7 vector (Clontech) and 10 μg library plasmid were cotransformed into the Y1H-Bait reporter strain. Then, the transformants were screened on the SD plates without uracil and leucine but with 900 ng/mL AbA (SD/-Ura/-Leu/AbA^900^ plates). They were cultured at 30°C for 3 days.

The positive clones were rescreened, picked for plasmid extraction, and transformed into E. coli DH5α for amplification, followed by DNA sequencing (General Biol Co., Ltd., Hefei, China). Finally, the target gene was mined based on the DNA sequence alignment and qRT-PCR analysis of the potential genes.

### Identification of positive interaction between GME12055 and the bait sequence in S. cerevisiae.

A Y1H-pAbAi strain was constructed by integrating the pAbAi plasmid (TaKaRa) into the Y1H genome and used as the control to verify whether the GME12055 could specifically bind to the bait DNA fragment. The pGADT7-12055 plasmid containing GME12055 cDNA was constructed and retransformed into the Y1H-Bait reporter strain or the Y1H-pAbAi strain. The transformation solution was gradient diluted, titrated (5 μL) on SD/-Ura/-Leu or SD/-Ura/-Leu/AbA^900^ plates, and incubated at 30°C for 3 days (61). A null control was also used by transforming the pGADT7 vector into the Y1H-Bait reporter strain.

### Sequence and phylogenetic analysis.

The amino acid sequences of SerRS homologs from 11 species were downloaded from UniProt (https://www.uniprot.org/). The multiple sequence alignment of ThserRS (accession no. OQ094962) and homologs was performed using Clustal Omega (https://www.ebi.ac.uk/Tools/msa/clustalo/). The phylogenetic tree was constructed using RaxML software (version 7.2.3) based on the maximum likelihood method.

### qRT-PCR analysis of genes at the transcriptional level.

The mycelia of *T. hirsuta* AH28-2 wild-type or mutant strain cultured in the XH liquid medium or on XH agar plates were withdrawn at indicated times for total RNA extraction. Then, 1 μg of total RNA was used as the template for cDNA synthesis following the PrimeScript RT kit (TaKaRa) instructions. The transcriptional levels of target genes were analyzed with qRT-PCR using an SYBR green kit (TaKaRa) and on a LightCycler 96 real-time PCR system (Roche, Basel, Switzerland). The gene *gapdh* was used as a reference gene to normalize the qRT-PCR data (46). The 2^−ΔΔCT^ method was used to calculate the relative expression levels of each gene (62). The primers for target genes, including *ThserRS*, *lacA*, *Trx*, *SOD*, *P450*, and *CAT*, are listed in Table S1.

### Construction of *ThserRS* overexpression, *gfp*-*ThserRS* overexpression, and *ThserRS* silencing *T. hirsuta* AH28-2 strains.

The *ThserRS* overexpression vector pYSK7-*Ov* and the silencing vector pYSK7-*An* were constructed based on the recombinant plasmid pYSK7, as previously described (32, 63). In particular, a 1,353-bp full-length cDNA and a 362-bp partial cDNA (from +398 to +759 bp) of *ThserRS* were amplified using the primer pairs of Ov-*ThserRS*-F and Ov-*ThserRS*-R and An-*ThserRS*-F and An-*ThserRS*-R, respectively (Table S1). The *gfp*-*ThserRS* overexpression vector pYSK7-*gfp*-*Ov* was constructed by fusing *egfp* to the full-length cDNA of ThserRS at the N terminus using primers *gfp*-*ThserRS*-F and *gfp*-*ThserRS*-R (Table S1). Then, they were inserted into plasmid pYSK7 through homologous recombination in the yeast S. cerevisiae Y1H. *T. hirsuta* AH28-2 spores were collected, treated with an enzyme solution to form protoplasts, and cotransformed the pYSK7-*Ov* vector, pYSK7-*gfp*-*Ov* vector, or pYSK7-*An* vector with the plasmid pCRII-*hph* mediated by the PEG/CaCl_2_ method (46, 64). Positive transformants were screened and further validated via genomic PCR amplification of the full-length cDNA of *ThserRS* or antisense *ThserRS* using primers PF and PR in Table S1 (46). Three *ThserRS* and *gfp*-*ThserRS* overexpression positive transformants and three *ThserRS* silencing positive transformants were randomly selected and performed for qRT-PCR analysis to quantify the transcriptional levels of *ThserRS* and *lacA*. Meanwhile, the laccase activity and growth characteristics of *ThserRS* overexpression and silencing transformants were analyzed.

### Laccase enzyme activity and native PAGE analysis.

Laccase activity was determined using guaiacol as the substrate, as previously described (30). Native PAGE was performed on 10% polyacrylamide gels according to the standard protocol. The gels were incubated in citrate-Na_2_HPO_4_ buffer (50 mM, pH 4.5) containing 2 mM guaiacol at 25°C for approximately 0.5 h (30). They were photographed using a digital camera. The intensity of laccase activity was calculated using ImageJ software (version 1.8.0).

### Mycelial growth rate assay.

*T. hirsuta* AH28-2 wild-type, *ThserRS* silencing, and *ThserRS* overexpressing strains were inoculated on XH agar plates with or without 100 μM CuSO_4_. They were incubated at 28°C in the dark for 5 to 8 days. H_2_O_2_ was added into the XH agar plates to final concentrations of 0, 5, 10, 15, 20, and 30 mM to evaluate the growth rates of strains. All colonies from the three parallel agar plates were photographed and measured. The pictures were transformed into grayscale maps. Then, the colony areas were calculated by pixel scale using Matlab software.

### Localization of ThserRS in yeast and *T. hirsuta* AH28-2.

The *egfp* was fused to the full-length cDNA of ThserRS at the N terminus. Then, the *gfp*-*ThserRS* fragment was introduced into a *Hin*d III/BamH I-digested yeast expression vector pYES2/CT under the control of the *GAL1* promoter. The recombined vector was transformed into the yeast S. cerevisiae Y1HGold using a lithium acetate method to generate the Y1H-GFP-ThserRS strain. This strain was spotted either on an SD-glucose plate or an SD-galactose plate with or without 100 μM CuSO_4_. It was incubated at 30°C for 3 days. Afterward, the cells were resuspended and fixed in 70% (vol/vol) ethanol, permeabilized, and stained with 1 μg/mL DAPI for 5 min in the dark following the manufacturer’s instructions.

The *gfp*-*ThserRS* overexpression *T. hirsuta* AH28-2 mycelia were grown on microscope slides with XH agar medium. Then, 100 μM CuSO_4_ was added for the Cu^2+^ induction group. The hyphae were fixed with cold methanol and stained with 2 μg/mL DAPI for 5 min in the dark. Then, the images were photographed using a laser confocal microscope (Olympus, Japan). All experiments were repeated at least three times.

### Structure modeling and molecular docking.

Human SerRS (PDB code: 3QNE), sharing 61.21% sequence identity with ThserRS, was used as the template for modeling the secondary and the 3D structures of ThserRS using the ESPript 3.0 online toolkit (https://espript.ibcp.fr/ESPript/cgi-bin/ESPript.cgi) and the Swiss Model (https://swissmodel.expasy.org). ThserRS homodimer was modeled via SymmetricDock (https://rosie.graylab.jhu.edu/symmetric_docking) (65). The interaction between ThserRS homodimer and the bait DNA fragment (−502 to −372 bp of the *lacA* promoter) was simulated on the HDOCK website (http://hdock.phys.hust.edu.cn/) (66). The result was visualized using the PyMOL software (version 2.5.2).

### Heterologous expression of ThserRS in E. coli and purification.

The full-length cDNA of *ThserRS* was amplified using specific primers (Table S1) and inserted into the pET28a (+) vector at sites *Nco* I and BamH I. The recombinant plasmid pET28a-*ThserRS* was transformed into E. coli BL21(DE3). The recombinant ThserRS was expressed by the addition of 1 mM isopropyl-β-d-thiogalactoside at a cell optical density at 600 nm of 0.8. It was incubated at 16°C and 120 rpm for 20 h. The cells were lysed in cold Tris buffer (40 mM, pH 7.8) containing 500 mM NaCl via sonication, followed by purification through Ni^2+^-NTA affinity chromatography (Novagen, Darmstadt, Germany). The protein purity was estimated by SDS-PAGE. The protein concentration was determined using the Bradford method.

### EMSA.

The 131-bp DNA fragment (−502 bp to −372 bp) and the truncated 32-bp DNA fragment of the *lacA* promoter (−462 bp to −431 bp) were synthesized in double-stranded forms (Sangon Biotech), end-labeled with 6-FAM at the 5′ terminus, and used as DNA probes. The probes (0.5 μM) were incubated with different concentrations of purified ThserRS in binding buffer A (25 mM HEPES [pH 6.5], 5 mM MgCl_2_, 150 mM KCl, 1 mM DTT, and 5% [vol/vol] glycerol) at 16°C for 30 min in a total reaction volume of 20 μL. Then, the mixtures were separated on a 6% natural PAGE in ice-cold 1× Tris-acetate-EDTA buffer (46). The DNA bands in the gels were visualized using a chemiluminescence imager (Smart Chemi 610; Sagecreation, Beijing, China). In addition, 100 ng poly d(I-C) or 10-fold identical unlabeled DNA fragments was added as nonspecific or specific competitors.

### FP assay.

The 40-nM FAM-labeled DNA probe was mixed with different concentrations of ThserRS in binding buffer A and incubated at 16°C for 30 min. The FP values of the reaction mixture were measured using a SpectraMax M5 instrument (Molecular Devices, San Jose, CA, USA) at excitation and emission wavelengths of 485 and 535 nm, respectively. The curves were visualized, and the dissociation constants (*K_d_*) were calculated using GraphPad Prism (version 9.0.0), according to Zhang et al. (46).

### Statistical analyses.

All experimental data are expressed as mean ± standard deviation. Statistical significance was evaluated in GraphPad Prism (9.0.0) using one-way ANOVA followed by Student’s *t* test, with the level set at *P < *0.05.
