# Supplementary material for: Seryl-tRNA Synthetase Shows a Noncanonical Activity of Upregulating Laccase Transcription in Trametes hirsuta AH28-2 Exposed to Copper Ion
Source: Microbiol Spectr. 2023 Jul 3;11(4):e00768-23. doi: 10.1128/spectrum.00768-23 (PMC10433817; doi:10.1128/spectrum.00768-23)
Supplement: Supplemental file 1 — Supplemental material. Download spectrum.00768-23-s0001.pdf, PDF file, 1.0 MB [file spectrum.00768-23-s0001.pdf]

**Seryl-tRNA synthetase shows a noncanonical activity of upregulating laccase transcription in *Trametes hirsuta* AH28-2 exposed to copper ion**

Zhiwei Gan[1,2,3#], Xueping Zhang[1,2,3#], Mengke Li[1,2,3], Xing Li[1,2,3], Xinlei Zhang[1,2,3], Chenkai Wang[1,2,3], Yazhong Xiao[1,2,3], Juanjuan Liu[1,2,3*], Zemin Fang[1,2,3*]

[1] School of Life Sciences, Anhui University, 230601 Hefei, Anhui, China

[2]Anhui Key Laboratory of Modern Biomanufacturing, 230601 Hefei, Anhui, China

[3]Anhui Provincial Engineering Technology Research Center of Microorganisms and Biocatalysis, 230601 Hefei, Anhui, China

[#] These authors contributed equally.

[*] Corresponding author

Phone/Fax: +86-551-63861861

Email: liu_juan825@ ahu.edu.cn (to JL), zemin_fang@ahu.edu.cn (to ZF).

## Supplementary Tables

### Table S1 Primers used in this study

| Name | Sequence (5'-3') | Purpose |
|---|---|---|
| Bait-F | ACGCGAGCTCTCCGTGAGAGGCCAG ATGTTGTTAG | Cloning of the bait DNA fragment |
| Bait-R | ACGCGTCGACGTGCCGGAGCTGGGC GCATGAG | Cloning of the bait DNA fragment |
| pAbAi-F | GTTCCTTATATGTAGCTTTCGACAT | Bait DNA validation |
| pAbAi-R | CATGTTAGGATGGGCAAGGCATTGA | Bait DNA validation |
| Ov-*ThserRS*-F | CTCCCATCTACACACAACAAGCTTATC GCCATGACTCTCGACGTCCTGCACTT | Cloning of *ThserRS* overexpression fragment |
| Ov-*ThserRS*-R | CACTGGCCCTCTGGTCAACTATAATAT TATCTATGCCTGTTTGCGCTGGAGC | Cloning of *ThserRS* overexpression fragment |
| An-*ThserRS*-F | CTCTGGTCAACTATAATATTATATCCCG AGGGCAAGGACGTGG | Cloning of *ThserRS* antisense fragment |
| An-*ThserRS*-R | ACACACAACAAGCTTATCGCCCCATT CATCCGAGTGGAAGGCG | Cloning of *ThserRS* antisense fragment |
| PF | ACATCCACCATCTCCGTTTTCTCCCAT | PCR of co-transformants |
| PR | TGACTATAGCAGCCTCCTACCACTG | PCR of co-transformants |
| *eGFP*-HindIII-F | CCCAAGCTTATGGTGAGCAAGGGCGA GGAGCTGTTCACC | Cloning of *eGFP* fragment (PYES2/CT vector) |
| *eGFP*-linker-R | AGAACCACTACCACTACCTGAACCCT TGTACAGCTCGTCCATGCCGAGAG | Cloning of *eGFP* fragment (PYES2/CT vector) |
| *ThserRS*-linker-F | GGTTCAGGTAGTGGTAGTGGTTCTAT GACTCTCGACGTCCTGCAC | Cloning of *ThserRS* fragment (PYES2/CT vector) |
| *ThserRS*-BamHI-R | CGCGGATCCCTATGCCTGTTTGCGCTG GTA | Cloning of *ThserRS* fragment (PYES2/CT vector) |
| T7-F | GGAATTCCATATGATGACTCTCGACG TCCTGCA | Cloning of *ThserRS* fragment (pGADT7 vector) |
| T7-R | CGCGGATCCCTATGCCTGTTTGCGCT GGT | Cloning of *ThserRS* fragment (pGADT7 vector) |
| *gfp*-*ThserRS*-F | CTCCCATCTACACACAACAAGCTTAT CGCCATGGTGAGCAAGGGCGAGGA | Cloning of *gfp*-*ThserRS* fragment (pYSK7 vector) |
| *gfp*-*ThserRS*-R | CACTGGCCCTCTGGTCAACTATAATA TTATCTATGCCTGTTTGCGCTGGT | Cloning of *gfp*-*ThserRS* fragment (pYSK7 vector) |
| *ThserRs*-NcoI-F | CATGCCATGGGCATGACTCTCGACGT CCTGCAC | Cloning of *ThserRS* fragment (pET28a vector) |
| *ThserRs*-BamHI-R | CGCGGATCCTTAGTGGTGGTGGTGGT GGTGTGCCTGTTTGCGCTGGTAGC | Cloning of *ThserRS* fragment (pET28a vector) |
| qRT-*ThserR*-F | GACTGGGTCAAGCTGGACTTCG | qRT-PCR of *ThserRS* |
| qRT-*ThserR*-R | GCGTTCTCTTTTGCCTTCTTTTTC | qRT-PCR of *ThserRS* |
| qRT-*lacA*-F | TCCTTCGTGTTGAATGCCGA | qRT-PCR of *lacA* |

| | | |
|---|---|---|
| qRT-*lacA* -R | GTTGATACCGCCCGCAAATC | qRT-PCR of *lacA* |
| qRT-*2703*-F | AAAGGGGGGAGGGTGCCCGTGC | qRT-PCR of *GME2703* |
| qRT-2703-R | GCGTCGGGGAAGATGTCGGCGA | qRT-PCR of *GME2703* |
| qRT-*4816*-F | CAGGGATCCCGTACCCCCAAGTATAC | qRT-PCR of *GME4816* |
| qRT-*4816*-R | CGGCAGCGACGGGATAGTTCAAG | qRT-PCR of *GME4816* |
| qRT-*Trx*-F | CGTGTATTCCGCCTACTGCCACCG | qRT-PCR of *Trx* |
| qRT-*Trx*-R | GGTGATAGCGGGTATCTTCCCGGT | qRT-PCR of *Trx* |
| qRT-*SOD*-F | CTTCCATATCCATGCCTCCGGCG | qRT-PCR of *SOD* |
| qRT-*SOD*-R | CGACCAACGATGCTCAACGGTCC | qRT-PCR of *SOD* |
| qRT-*CAT*-F | CACGATGATGGACATGCTGAGCG | qRT-PCR of *CAT* |
| qRT-*CAT*-R | GGTGGCGCTTCTGCGTATGAATG | qRT-PCR of *CAT* |
| qRT-*CYP450*-F | CTTCAGCACACCACGGTTGTTGACC | qRT-PCR of *CYP450* |
| qRT-*CYP450*-R | GGCGGTGTTGGTCGCCTTCCGTA | qRT-PCR of *CYP450* |
| qRT-*gapdh*-F | GCCGCTTCAAGGGCAAAGTC | qRT-PCR of *gapdh* |
| qRT-*gapdh*-R | TGTAGTCGGCACCAACGGA | qRT-PCR of *gapdh* |

**Table S2 Conserved nucleotides and positions of XREs in four antioxidant genes**

| | TCACGC | TGCGTG | TCATGC | TNGCGTG |
|---|---|---|---|---|
| CYP450 | -1596 bp | / | / | / |
| Trx | -1532 bp | / | / | / |
| CAT | / | -1734 bp; -1595 bp | -265 bp | / |
| SOD | / | -1498 bp | / | -952 bp |

## Supplementary Figures

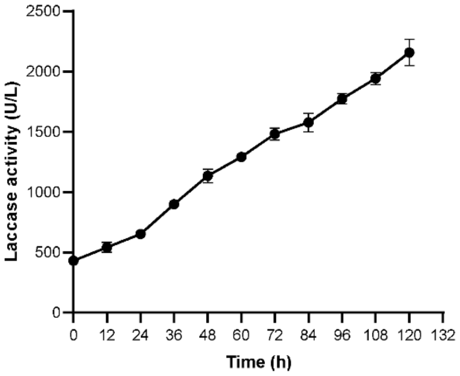


**Figure S1 Laccase activity of *T. hirsuta* AH28-2 in fermentation with copper induction.** *T. hirsuta* AH28-2 was cultured in XH liquid medium added with 100 μM $CuSO_4$.

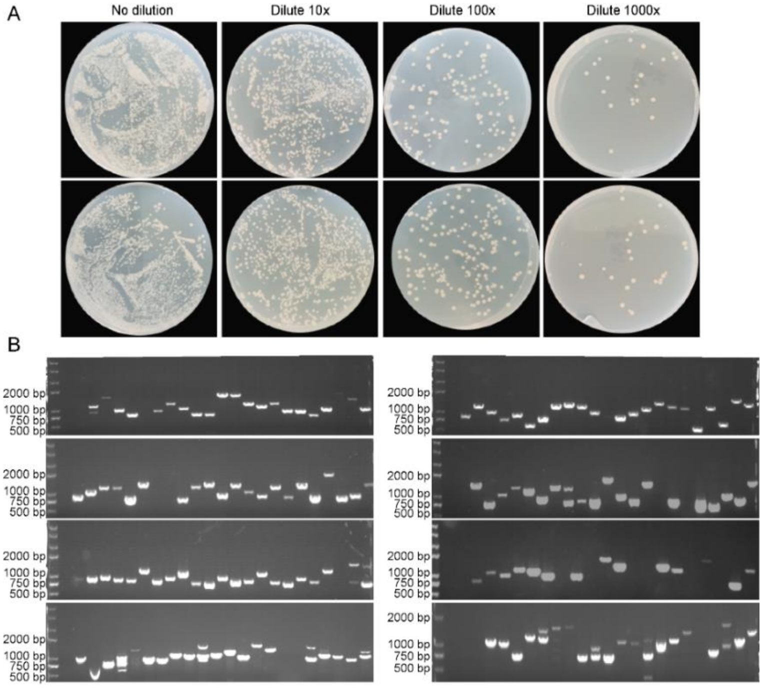


**Figure S2 Screening of *lacA* promoter binding proteins.** (A) Transformation efficiency was calculated for a plate diluted 1000 times. 100 μL of transformation solution produced 26 monoclonal clones on average and the total transformation system

was 15 ml. 26/0.1×1000×15= 3.9 million. (B) The PCR results of clones grow on SD/-Ura/-Leu/AbA$^{900}$ plates.

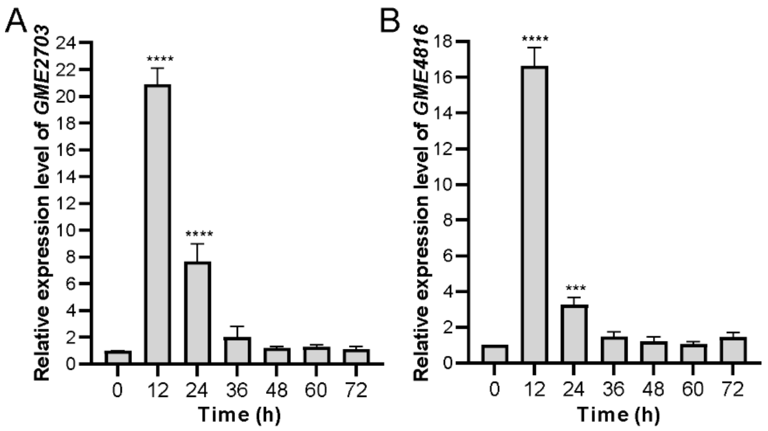


**Figure S3 *GME2703* (A) and *GME4816* (B) are substantially increased at 12 h but sharply decreased afterwards in *T. hirsuta* AH28-2 upon Cu$^{2+}$ induction.** *T. hirsuta* AH28-2 mycelia exposed to 100 μM $CuSO_4$ were collected every 12 h, extracted for RNA and analyzed the transcripts of *GME2703* and *GME4816* by qRT-PCR. The transcriptional levels of *GME2703* and *GME4816* at 0 h were set as baselines. The data were analyzed using a student's *t*-test as compared with the WT strain (**P* < 0.05, ***P* < 0.01, ****P* < 0.001). Data show mean ± standard deviation, n = 3.

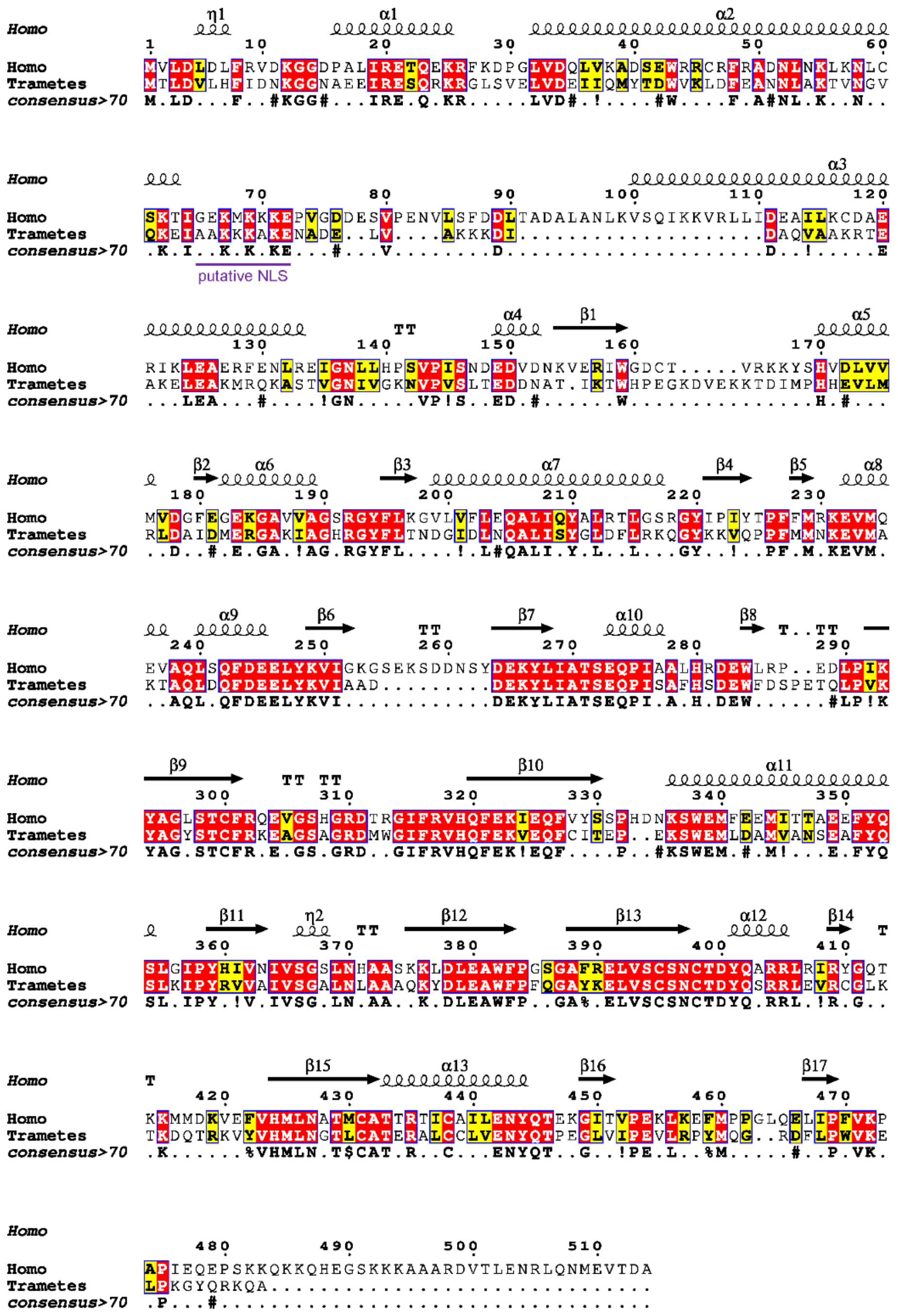


**Figure S4 The secondary structure prediction of ThserRS using human SerRS as the template.** The putative NLS ($AAKKKAKE_{65-72}$) was showed.

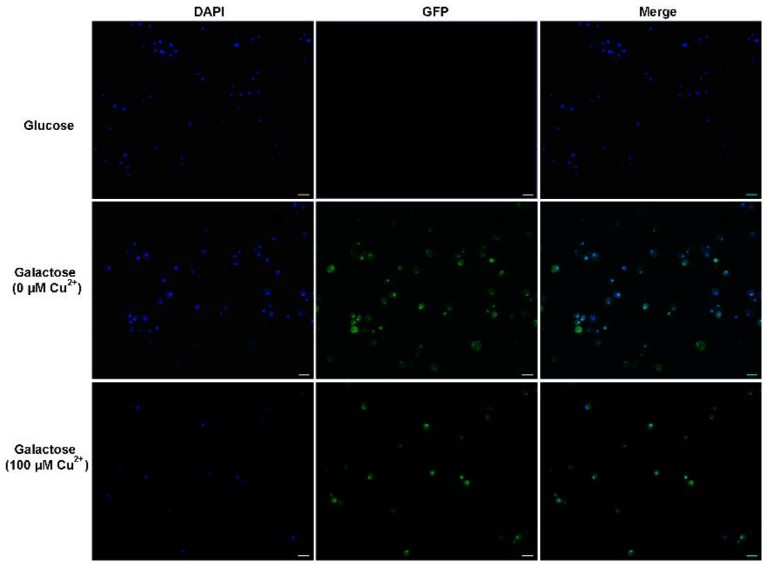


**Figure S5 ThserRS is partially localized in nucleus in yeast.** Scale bars, 10 μm.

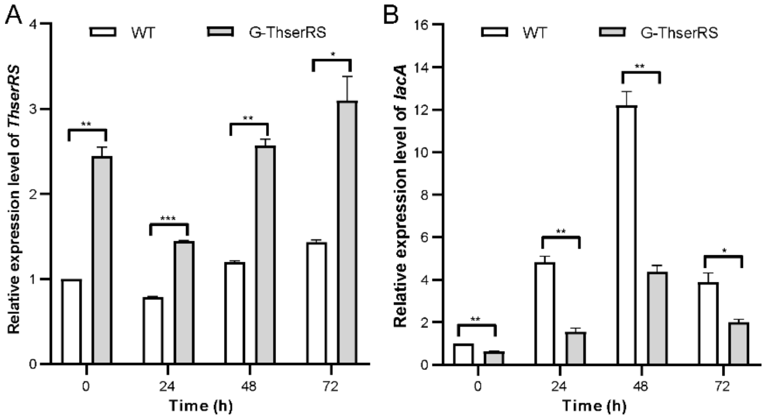


**Figure S6 The GFP fusion does not interfere with the function of ThserRS on *lacA* transcription in strain G-ThserRS.** The mycelia of *GFP-ThserRS* overexpression *T. hirsuta* AH28-2 strain named G-ThserRS exposed to 100 μM $CuSO_4$ were collected every 24 h, extracted for RNA and analyzed the transcripts of *ThserRS* and *lacA* by qRT-PCR. The transcriptional levels of *ThserRS* and *lacA* in the WT strain at 0 h were set as baselines, respectively. The data were analyzed using a student's *t*-test as compared with the WT strain (*$P < 0.05$, **$P < 0.01$, ***$P < 0.001$). Data show mean ± standard deviation, n = 3.

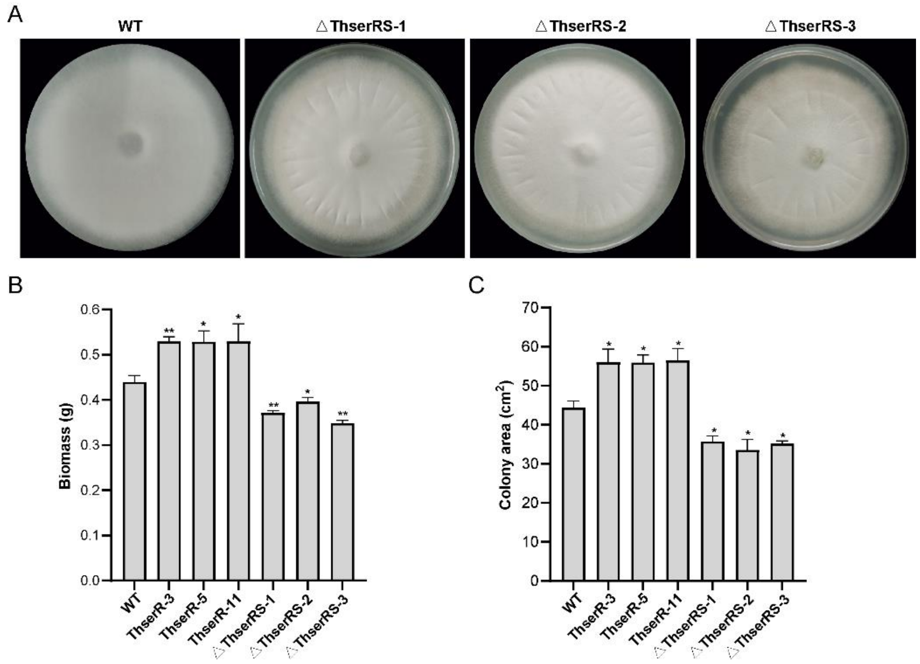


**Figure S7 ThserRS is involved in mycelia growth.** (A), *ThserRS* silencing affects the colony phenotype under $Cu^{2+}$ treatment. The wild-type (WT) and *ThserRS* silencing *T. hirsuta* AH28-2 strains were incubated on XH agar plates adding 100 μM $CuSO_4$ for 8 d in a dark incubator at 28 °C. (B, C), *ThserRS* overexpressing accelerates but silencing reduces the biomasses of *T. hirsuta* AH28-2 in liquid media (B) or the mycelia expansion rate on agar plates (C) without $Cu^{2+}$ addition. The seven strains cultured in liquid XH medium without $CuSO_4$ were collected at 120 h and they were incubated on XH agar plates without $CuSO_4$ for 5 days in a dark incubator at 28 ℃. The data were analyzed using a student's *t*-test as compared with the WT strain (*$P < 0.05$, **$P < 0.01$). Data show mean ± standard deviation, n = 3.

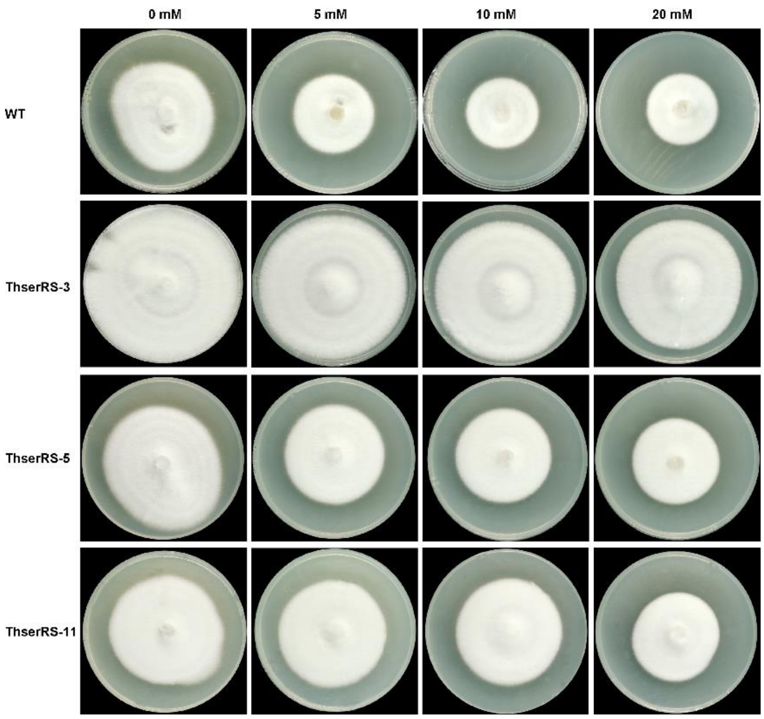


**Figure S8 *ThserRS* overexpressing decreases the strains sensitivity to $H_2O_2$.**
